# Multiple evolutionary lineages for the main vector of *Leishmania guyanensis, Lutzomyia umbratilis* (Diptera: Psychodidae), in the Brazilian Amazon

**DOI:** 10.1038/s41598-021-93072-4

**Published:** 2021-07-28

**Authors:** Vera Margarete Scarpassa, Antônio Saulo Cunha-Machado, Ronildo Baiatone Alencar

**Affiliations:** 1 Laboratório de Genética de Populações e Evolução de Mosquitos Vetores, Coordenação de Biodiversidade, Instituto Nacional de Pesquisas da Amazônia, Avenida André Araújo, 2936. Bairro Aleixo, Manaus, Amazonas CEP 69.067-375 Brazil; 2 Laboratório de Biologia Molecular, Centro de Biotecnologia da Amazônia, Avenida Governador Danilo Areosa s/n. Distrito Industrial I, Manaus, Amazonas CEP 69.075-351 Brazil; 3 Departamento de Vigilância Epidemiológica, Fundação de Vigilância em Saúde do Estado do Amazonas, Manaus, Amazonas CEP 69.093-018 Brazil

**Keywords:** Evolution, Genetics

## Abstract

*Lutzomyia umbratilis* is the main vector of *Leishmania guyanensis* in the Brazilian Amazon and in neighboring countries. Previous biological and molecular investigations have revealed significant differences between *L. umbratilis* populations from the central Brazilian Amazon region. Here, a phylogeographic survey of *L. umbratilis* populations collected from nine localities in the Brazilian Amazon was conducted using two mitochondrial genes. Statistical analyses focused on population genetics, phylogenetic relationships and species delimitations. *COI* genetic diversity was very high, whereas *Cytb* diversity was moderate. *COI* genealogical haplotypes, population structure and phylogenetic analyses identified a deep genetic differentiation and three main genetic groups. *Cytb* showed a shallower genetic structure, two main haplogroups and poorly resolved phylogenetic trees. These findings, allied to absence of isolation by distance, support the hypothesis that the Amazon and Negro Rivers and interfluves are the main evolutionary forces driving *L. umbratilis* diversification. The main three genetic groups observed represent three evolutionary lineages, possibly species. The first lineage occurs north of the Amazon River and east of Negro River, where *Le. guyanensis* transmission is intense, implying that *L. umbratilis* is an important vector there. The second lineage is in the interfluve between north of Amazon River and west of Negro River, an area reported to be free of *Le. guyanensis* transmission. The third lineage, first recorded in this study, is in the interfluve between south of Amazon River and west of Madeira River, and its involvement in the transmission of this parasite remains to be elucidated.

## Introduction

The diversification patterns (genetically structured populations, evolutionary lineages, complete speciation) in phlebotomine Diptera have been linked to multiple factors, such as climate events, geographic barriers (rivers, mountains), biomes, latitude and altitude, habitat modification and landscape fragmentation caused by anthropogenic actions, among others^1^. Such factors may reduce the dispersal capacity of sandflies, leading them to become isolated populations, with a resulting loss of genetic diversity within populations and the increase in differentiation between them^1–5^. The low flight capacity^6,7^, characteristic of these insects, and the type of soil used for breeding are also factors that may contribute further to population isolation in consequence favor genetic differentiation and, eventually, speciation. In this context, studying the microevolutionary processes that act at the level of sandflies vector populations is key for the identification of these patterns, which are baselines for the epidemiological studies, surveillance and vector control strategies.

It has been proposed that the major rivers of the Amazonian region have acted, and continue to act, as significant dispersal barriers for a variety of taxa, including primates^8^, lizards^9^, frogs^10^, birds^11,12^ and insects^13,14^—a scenario referred to as the “river-barrier hypothesis”^15^. This hypothesis predicts that major Amazonian rivers significantly reduce or prevent gene flow between populations inhabiting opposite banks, so promoting genetic divergence and increasing the opportunity for allopatric speciation. If this is correct, (1) it is expected that sister species will occur on opposite river banks, (2) the genetic similarity between populations will be greater in sites on the same bank that in sites on opposite banks separated by the same distance, and (3) the boundaries of species distributions will coincide with large rivers^16,17^.

*Lutzomyia* (*Nyssomyia*) *umbratilis* Ward and Fraiha, 1977 is the main vector of the etiologic agent that causes American Cutaneous Leishmaniasis (ACL), *Leishmania* (*Viannia*) *guyanensis* Floch, 1954, in north South America^18,19^. In the Brazilian Amazon, *L. umbratilis* is highly anthropophilic and has been identified as the main vector of this parasite in the states of Amazonas, Amapá, Pará and Roraima^20,21^, an area that together holds > 50% of the cases of human leishmaniasis recorded for the whole Brazilian Amazon^21^. *Lutzomyia umbratilis* has also been implicated in the transmission of *Le. guyanensis* in French Guiana and Suriname^18–20,22,23^.

This phlebotomine species occurs in northern South America, including Bolivia, Brazil, Colombia, French Guiana, Guyana, Peru, Suriname and Venezuela^24,25^. In Brazil, *L. umbratilis* has been recorded in all states in the northern region^19,20,22–24^, as well as the states of Mato Grosso and Mato Grosso do Sul (west central and southwest regions)^26,27^, and the states of Maranhão, Pernambuco and Alagoas, in the northeastern region^24,28–30^. This vast geographic area, with discontinuous distribution and the presence of geographic barriers, along with the low flight capacity of these insects^6,7^, means that *L. umbratilis* populations could be highly susceptible to evolve into differentiated populations, evolutionary lineages and distinct species.

*Lutzomyia umbratilis* was described by Ward and Fraiha^31^, based on specimens (females) captured in the Monte Dourado area of the Jari River (north of Amazon River), state of Pará, Brazil. Prior to this *L. umbratilis* had been wrongly identified as *Lutzomyia anduzei* Rozeboom 1942^32^ due to their great morphological similarity. The species have overlapping geographic distributions across a large portion of northern South America^24^. Nowadays, they may be distinguished based on the internal and external genitalia morphology of male and female adults and by molecular markers^24,33^.

In the Brazilian Amazon, adults of *L. umbratilis* are often found in rainforest, especially in areas of the high humidity and dim light, as consequence, this species has been recognized as ombrophilous, as is expressed in its name – *L. umbratilis*. Adults of this species have been captured using aspirators in bases of tree trunks during daytime, as well as with CDC (Center for Disease Control) miniature light traps at ground level, and in the forest canopy at night – a behavior termed acrodendrophily^34^. *Lutzomyia umbratilis* seems to be abundant in the central Brazilian Amazon region, tending to decline towards the margins of this region^35^.

Arias and Freitas^13,36^ reported that the susceptibility of this vector to *Leishmania* may vary within the central Brazilian Amazon region. These authors reported *L. umbratilis* populations naturally infected with *Le. guyanensis* to the north of the Negro and Amazon Rivers; but they did not observe natural infections by *Leishmania* in this species to the north of the Amazon River and west of the Negro River. Arias and Freitas^13^ hypothesized that this fluvial system could act as a vicariant barrier to *Le. guyanensis* transmission. In line with this hypothesis^13^, a study of experimental infection, performed with two samples of *L. umbratilis* from opposite banks of these rivers, revealed higher vector competence in a sample from north of Amazon River than in sample from north of the Amazon River and west of the Negro River^37^, supporting the Arias and Freitas’s hypothesis^13^. In fact, the presence of cryptic or sibling species complexes in a given region can produce heterogeneous patterns of parasite transmission, because they may differ in vector competence (the intrinsic ability of a vector to transmit a pathogen), host feeding preference, feeding behavior and breeding sites as well as they may either occur sympatrically or have distinct geographical distributions.

A biological study conducted with *L. umbratilis* populations from Manaus and Manacapuru (north of Amazon River/east of Negro River and north of Amazon River/west of Negro River, respectively) revealed significant biological differences^38^. A second study that combined morphology and isozymes analyses of four *L. umbratilis* populations from this fluvial system showed subtle morphological differences in the immature stage and in female genitalia, whereas the isozymes markers did not reveal any differences among populations studied^39^. However, isozymes are not appropriated markers to separate species that have recently diverged.

Unlike the results with isozymes, a phylogeographic structure study using molecular markers (*COI-*mtDNA) revealed low and non-significant differentiation between populations situated on the same banks of the Amazon and Negro Rivers, but marked and very significant differences between populations situated on the opposite banks of these rivers^33,35,40^. This pattern resulted in the absence of isolation by distance (IBD). Although all haplotypes were connected in a unique network, the strong bimodality observed with the mismatch analyses, when all samples were analyzed together, was consistent with long-term isolation, suggesting historical isolation between the analyzed populations^40^. These groups probably attained complete isolation following the most recent formation of the Negro and Amazon Rivers network (~ 2.4 Mya to present), which was considered as the main evolutionary force. In line with these results, another study, using the Barcode region (*COI*) of these *L. umbratilis* two groups, also failed to find evidence for haplotype sharing. However, they could be identified by unique fixed mutation, as well as the genetic distance was rather small^33^. Later, Freitas and others^41^, using also a *COI* fragment, compared Manacapuru and Rio Preto da Eva populations, situated on the opposite banks of the same rivers, with a sample of Recife, in the northeast region from Brazil. Consistent with previous studies^33,40^, two distinct groups were observed: one group clustered individuals from Rio Preto da Eva (north Amazon River and east Negro River) plus Recife and, another group, clustered only individuals of Manacapuru (interfluve located of the north Amazon River and west of Negro River). This finding indicates that the Recife population may have been derived from populations from the north Amazon River.

However, most of these studies are based on samples from a small number of locations. As a result, additional investigations are needed to fully elucidate the number of species in this complex and the role of each one in the transmission of *Le. guyanensis* in the Amazonian region. Such information provides an essential baseline for epidemiology, as well as for surveillance and vector control measures. In this context, the current study investigated the molecular variation and phylogeographic structure of *L. umbratilis* populations collected from nine geographic areas in the Brazilian Amazon region, inferred by sequencing of two mitochondrial fragments (*COI* and *Cytb* genes) to test the hypotheses: (1) Are the largest rivers in the Brazilian Amazon acting as barriers on the *L. umbratilis* populations? (2) Are the populations located on the opposite banks of the Amazon and Negro Rivers and in the interfluves genetically different? (interfluves = beard or the elevated area between two watercourses or two valleys); (3) How many lineages or species exist within *L. umbratilis* complex in the region studied?

## Material and methods

### Sandfly collection and morphological identification

**Figure 1:**
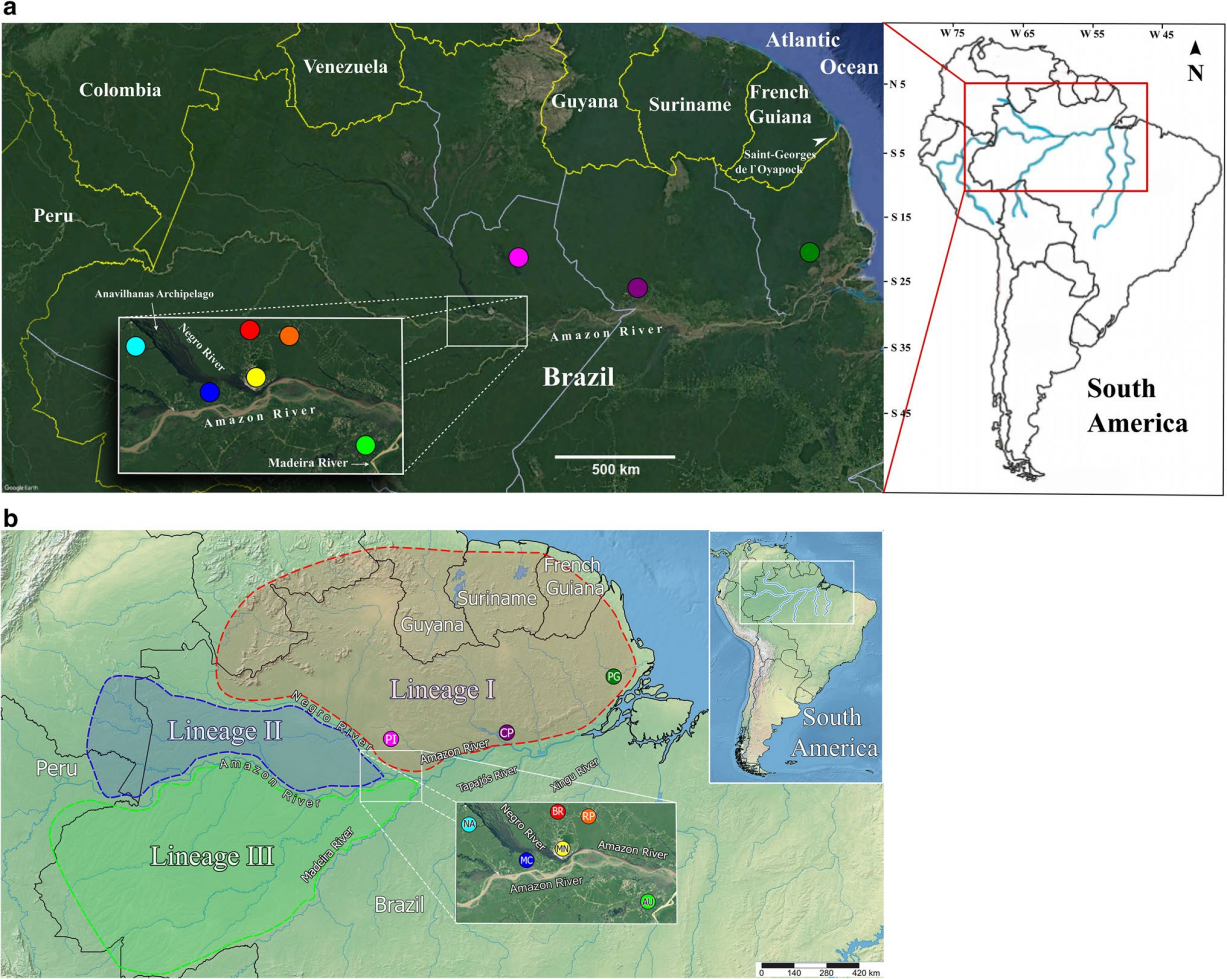
(**a**) Collection sites of the nine *Lutzomyia umbratilis* populations from the Brazilian Amazon and the geographic distribution of three lineages inferred in this study. The colored circles on the map represent the sampled sites. Purple: Cachoeira Porteira (CP); Red: km 43 of BR-174 Highway (BR); Orange: Rio Preto da Eva (RP); Yellow: Manaus (MN); Dark blue: Manacapuru (MC); Light blue: Novo Airão (NA); Pink: Pitinga (PI); Light green: Autazes (AU); Dark green: Porto Grande/Serra do Navio (PG). Map was created using SimpleMappr (https://www.simplemappr.net) (public domain) and adapted for illustrative purposes in Inkscape version 1.0.1 (https://www.inkscape.org). (**b**) Geographic distribution inferred for the three lineages of this study. Lineage I (Red); Lineage II (Blue), Lineage III (Green). Map was created using SimpleMappr (https://www.simplemappr.net) (public domain) and adapted for illustrative purposes in Inkscape version 1.0.1 (https://www.inkscape.org).

Collection of *L. umbratilis* adults were performed in nine localities within the Brazilian Amazon, with seven localities in the state of Amazonas (km 43 of BR-174 Federal Highway [BR]; km 65 of AM-010 Highway in the municipality of Rio Preto da Eva [RP]; Manaus [MN]; km 60 of AM-070 Highway in the municipality of Manacapuru [MC]; km 60 and km 70 of AM-352 Highway in the municipality of Novo Airão [NA]; Pitinga [PI]; Ramal do Sampaio in the municipality of Autazes [AU]), one in state of Pará (municipality of Oriximiná, Cachoeira Porteira [CP]), and one in state of Amapá (Porto Grande/Serra do Navio [PG]). Figure 1a shows the locations of all nine study sites: (1) north of Amazon River and east of Negro River**:** Cachoeira Porteira (CP; purple), km 43 of BR-174 Highway (BR, red), Rio Preto da Eva (RP, orange), Manaus (MN, yellow), Pitinga (PI, pink) and Porto Grande/Serra do Navio (PG, dark green); (2) north of Amazon River and west of Negro River (interfluve 1, between Amazon and Negro Rivers): Manacapuru (MC, dark blue) and Novo Airão (NA, light blue); (3) south of Amazon River and west Madeira River (interfluve 2, between Amazon and Madeira Rivers): Ramal do Sampaio, in the municipality of Autazes (AU, light green). Table 1 provides information on the localities sampled, geographic co-ordinates and sample sizes for each marker.

**Table 1:** Information on the localities, geographic co-ordinates and sample size for each marker analyzed in *Lutzomyia umbratilis* from the Brazilian Amazon.

Locality, State	Co-ordinates		Sample size	
	Latitude	Longitude	*COI*	*Cytb*
Cachoeira Porteira, Pará*	01° 28′ S;	56° 22′ W	18*	**25**
Km 43 of BR-174 Highway, Amazonas*	02° 36′ S;	60° 02′ W	15*	**17**
Rio Preto da Eva, Amazonas*	02° 43′ S;	59° 47′ W	15*	**25**
Manaus, Amazonas**	03° 04′ S;	59° 57′ W	22**	**25**
Manacapuru, Amazonas*	03° 14′ S;	60° 31′ W	24*	**30**
Novo Airão, Amazonas*	02° 47′ S;	60° 55′ W	35*	**24**
Pitinga, Amazonas	00° 47′ S;	60° 08′ W	**34**	**29**
Autazes, Amazonas	03° 41′ S;	59° 07′ W	**04**	**03**
Porto Grande/Serra do Navio, Amapá	00° 42′ N;	51° 25′ W	**09**	**09**
**Total**			**176**	**187**

*COI* = Cytochrome Oxidase, subunit I; *Cytb* = Cytochrome b; * = populations analyzed by Scarpassa and Alencar^40^; ** = sample size was enlarged and re-analyzed in the present study. The numbers in **bold** are the specimens analyzed in the present study.

The *COI* sequences of the individuals from Cachoeira Porteira, km 43 of BR-174 Highway, Rio Preto da Eva, Manaus, Manacapuru and Novo Airão were previously analyzed by Scarpassa and Alencar^40^, while three new samples (Pitinga, Autazes and Porto Grande/Serra do Navio) were analyzed in this study using the same *COI* fragment (Table 1). Additionally, in the current study, the sample size of Manaus was expanded, with 18 new specimens sequenced, totaling n=22, and re-evaluated (Table 1). This database was integrated with Scarpassa and Alencar’s data^40^ and re-evaluated in this study. For the *Cytb* gene, nine populations were analyzed in the current study (Table 1).

Sandflies were collected with CDC miniature light traps (CDC “miniature”- HAUSHERR MACHINE WORKS, NEW JERSEY, EUA)^42^, and also with aspirators on the bases of trunks of rainforest trees during the daytime. To collect a random population sample and avoid an excess of offspring from the same females, we followed Scarpassa and Alencar^40^ and sampled a minimum of five tree trunks within a given study area. Captured specimens were killed at – 20 °C freezer and morphologically identified based on genital morphology, following the taxonomic key of Young and Duncan^24^ and the characters described in Scarpassa and Alencar^33^. Following identification, specimens were preserved in 95% ethanol and stored at – 20 °C until genomic DNA was extracted. Sample collections were made under the System of Information on the Biodiversity (SISBIO), using permanent license number 38440-1 awarded to VMS.

### Genomic DNA extraction, PCR amplification and sequencing of the COI and Cytb markers

For the *COI* gene, 176 specimens were analyzed, with 111 from the study of Scarpassa and Alencar^40^, and 65 analyzed specifically for the current study. For the *Cytb* gene, 187 specimens from the nine localities were analyzed in this study (Table 1).

Total genomic DNA was extracted individually from whole sandflies using the Phenol–Chloroform protocol^43^, with the DNA pellet resuspended in 30 μL of 1xTE buffer (10 mM Tris-Cl pH 8.0; 1 mM EDTA pH 8.0) or in sterile water. A small aliquot of this DNA was stored at − 20 °C and used in the amplification by polymerase chain reaction (PCR) of the two analyzed markers. All DNA remaining from the specimens was kept frozen at − 81 °C as voucher DNA in the vector insect collection at the Laboratory of Population Genetics and Evolution of Vector Mosquitoes at the Instituto Nacional de Pesquisas da Amazônia, in Manaus, Brazil. DNA extracted from all individuals was amplified with specific oligonucleotide primers for the two genes, as described below.

A long fragment (1181 bp), representing the variable region of end 3’ of cytochrome c oxidase I gene (*COI*), was amplified by PCR using the primers described in Zhang and Hewitt^44^. The PCR reactions was included Platinum Taq DNA Polymerase of High Fidelity (LIFE TECHNOLOGY), and negative control was included for all reactions. The PCR products were electrophoresed in 1% agarose gel stained with Gel Red Nucleic Acid Gel Stain (BIOTIUM INC., HAYWARD, USA) and visualized under UV light to check the size and quality of expected products. The amplified products were purified using PEG precipitation (20% polyethylene glycol 8000 and 2.5 M NaCl). Both DNA strands were sequenced using the Big Dye Terminator Kit, v. 3.1. The reactions were electro-injected in an automated ABI 3130 xl Genetic Analyzer (APPLIED BIOSYSTEMS, THERMO FISHER SCIENTIFIC, WALTHAM, MA, USA).

A fragment of 512 bp from the *Cytb* gene was amplified by PCR using the primers described in Coutinho-Abreu and others^45^. The PCR reactions was included Platinum Taq DNA Polymerase of High Fidelity (LIFE TECHNOLOGY), and a negative control was used in all reactions. The PCR products were electrophoresed in 1% agarose gel stained with Gel Red Nucleic Acid Gel Stain (BIOTIUM INC., HAYWARD, USA), and visualized under UV light to verify the size and quality of expected products. Amplified products purification and sequencing reactions were carried out as described above.

### Statistical analyses

*COI* and *Cytb* sequences were automatically aligned using Clustal W, and manually edited in BioEdit v. 7.0.8.0^46^ with the aid of the electropherogram viewer Chromas Lite^47^. Consensus sequences of both genes were confirmed with the mitochondrial genome of *L. umbratilis*^48^, using the BLAST (BASIC LOCAL ALIGNMENT SEARCH TOOL)^49^, which resulted in 99 to 100% identity. The sequences of this study were mapped between the positions 1759 and 2939 bp for *COI*, and between the positions 11,241 and 11,752 bp for *Cytb* of mitochondrial genome of this species^48^.

Both *COI* and *Cytb* datasets were checked for saturation levels against genetic distances using DAMBE^50^, which allows testing for the presence of any saturation between transition and transversion rates in relation to genetic distances. Results showed no saturation for either marker, indicating the datasets are appropriate for the applied phylogenetic inferences. Haplotypes were determined using DnaSP v. 5.10^51^ and TCS v. 1.21^52^. Identical sequences were considered to represent a single haplotype. To analyze population history and haplotype genealogy, haplotype networks were analyzed for the two markers separately. To calculate the maximum number of mutational connections between pairs of sequences, the 95% parsimony criterion was used, with analyses run on TCS v. 1.21^52^. Using this criterion, the possibility that all samples analyzed formed a single network or two or more disconnected networks was investigated.

Unfortunately, concatenated analyses with two markers were not possible as the DNAs of the same individuals from Manacapuru and Novo Airão populations did not amplify for both markers used. Thus, the phylogenetic relationships were inferred with all haplotypes for each marker using Neighbor-Joining (NJ) and Maximum Likelihood (ML) in MEGA v. 7.0^53^ and Bayesian Inference (BI) with the Mr.Bayes v. 3.2.5^54^ and *BEAST v. 2.0^55^ algorithms. Kimura2-Parameters (K2P) distances^56^ were used for the NJ analysis, with 5000 replicates, while the ML and BI analyses were inferred using the General Time Reversible (GTR) + G + I nucleotide substitution model for *COI*, and the HKY nucleotide substitution model for *Cytb*, both previously selected with the Akaike Information Criterion (AIC) via jModelTest^57^. In the ML analyses, a tree was generated with 2000 replicates. In the BI analyses, for each marker two simultaneous independent runs of the Markov Chain Monte Carlo (MCMC) were performed for 100 million generations, while sampling every 1000 generations with a burn-in of 25%. Bayesian Posterior Probabilities (BPP) were used to assess nodal support.

To infer the limits between lineages, three models based on single locus data (*COI*) were used: GMYC (Generalized Mixed Yule Coalescent)^58^, bGMYC (with implementation Bayesian of GMYC)^59^ and ABGD (Automatic Barcode Gap Discovery)^60^. Delimitation with the GMYC approach is based on assigning branching events to two categories, speciation and coalescent within species. With the assumption that species are monophyletic, a set of most recent common ancestor nodes can be specified that determines the type of branching events. bGMYC model identifies uncertainties in the limits of the species as per the changes in the ramification rates in the phylogenetic tree when distinct populations contain several species. ABGD model allows partitioning of the DNA sequence datasets into clusters of similar taxa, establishing a range of maximum values of intraspecific divergence (P), without an a priori species hypothesis. These analyses were conducted using the bGMYC SPLITS package^61^ and implemented in the R package, version 3.6.0^61^. The ultrametric trees were generated with *BEAUTi and *BEAST v.1.7^62^ and used in these analyses.

The divergence time of the groups (lineages) were also estimated in *BEAST v. 2.0^55^, using a relaxed lognormal clock with a Yule tree prior, assuming a constant lineage birth rate for each tree branch, and with a mutation rate of 2.3% for every million years for the *COI* gene^63^.

All phylogenetic analyses also included fragments corresponding to the *COI* and *Cytb* regions extracted from mitochondrial genome of *L. umbratilis* from Saint-Georges de l’Oyapock, French Guiana [Accession numbers: KP702938 and KP702939]^48^. Saint-Georges de l’Oyapock is situated on the border with the state of Amapá, Brazil, and lies north of the Amazon River (Fig. 1a). *Lutzomyia anduzei* and *Bichromomyia flaviscutellata* were used as outgroups in these analyses. Tree topologies were visualized and edited in FigTree v. 1.3.1^64^.

Principal Coordinates Analysis (PCA) was also implemented for the *COI* using the R package, *spider* 1.5.0 with the function *ordinDNA*^65^. In this analysis, the distance between the clusters is approximately proportional to the genetic distances between species.

### Genetic diversity and population structure

The intra-population genetic diversity measures, such as haplotype (*h*) and nucleotide (*π*) diversities, the average number of nucleotide differences (*K*), number of polymorphic sites (*NS*), transition and transversion rates (*T*s/*T*v), and the neutrality tests of Tajima’s *D*^66^ and Fu’s *F*s tests^67^ for each sample and total were inferred using DnaSP, v. 5.10^51^ and Arlequin, v. 3.1^68^. Tajima’s *D* was run to test strict neutrality, while the neutrality test, Fu’s *F*s, was estimated to test population size stability. The latter test is more powerful for detecting population expansion and genetic hitchhiking.

Using the *F*_ST_ statistic, genetic differentiation between samples was inferred in Arlequin, v. 3.1^68^. The average number of nucleotide substitutions per site between samples (*D*_*xy*_), the number of net nucleotide substitutions per site between samples (*D*_*a*_), the number of shared polymorphisms between samples pairs (*S*_*s*_), and a number of fixed differences between samples pairs (*S*_*f*_) were calculated in DnaSP, v. 5.10^51^.

To check the number of genetic groups obtained in the previous analyses, we implemented the Bayesian Analysis of Population Structure (BAPS) with BAPS software^69,70^ for both markers. In these analyses, all sequences from both datasets were used and 1–9 clusters were employed (the upper corresponding to the total number of sampled localities), and five independent runs were made. The most probable genetic cluster configuration was prepared by comparing the log-likelihood values of the best models.

The genetic groups retrieved in the haplotypes network and phylogenetic analyses were used to estimate the mean intra- and inter-group genetic distances using the uncorrected-*p* genetic distance. These analyses were calculated in MEGA, v. 7.0^53^, and the standard errors were estimated by bootstrapping with 1000 replicates.

To test the isolation by distance (IBD) hypothesis, the correlation between straight-line geographic distances and *F*_ST_ values among samples was applied using a Mantel test^71^ in Arlequin, v. 3.1^68^ with 2000 permutations. Straight-line geographic distances among the localities were estimated using Google Earth.

## Results

The molecular variation and phylogeographic structure of nine natural populations of *L. umbratilis* from the Brazilian Amazon were estimated. A total of 176 specimens were analyzed for *COI* and 187 for *Cytb*, totaling 363 individuals sequenced (Table 1). Fragment sizes were 1181 bp and 512 bp for *COI* and *Cytb*, respectively, totaling 1693 bp. All alignments were unambiguous and no insertions or deletions were detected in either dataset. Transitions (89.68%; 83.33%) were more common than transversions (10.32%; 16.67%), with most occurring at the third codon position (all synonymous) for both markers. *COI* and *Cytb* had 110 (9.31%) and 41 (8.01%) variable sites, of which 56 (4.74%) and 14 (2.73%) were parsimoniously informative, respectively. The average nucleotide composition for the *COI* was 39.9% T, 30.7% A (A + T = 70.6%), 15.2% C, and 14.1% G, while for *Cytb* these values were 39.5% T, 38.5% A (A + T = 78%), 13.5% C, and 8.5% G.

**Figure 2:**
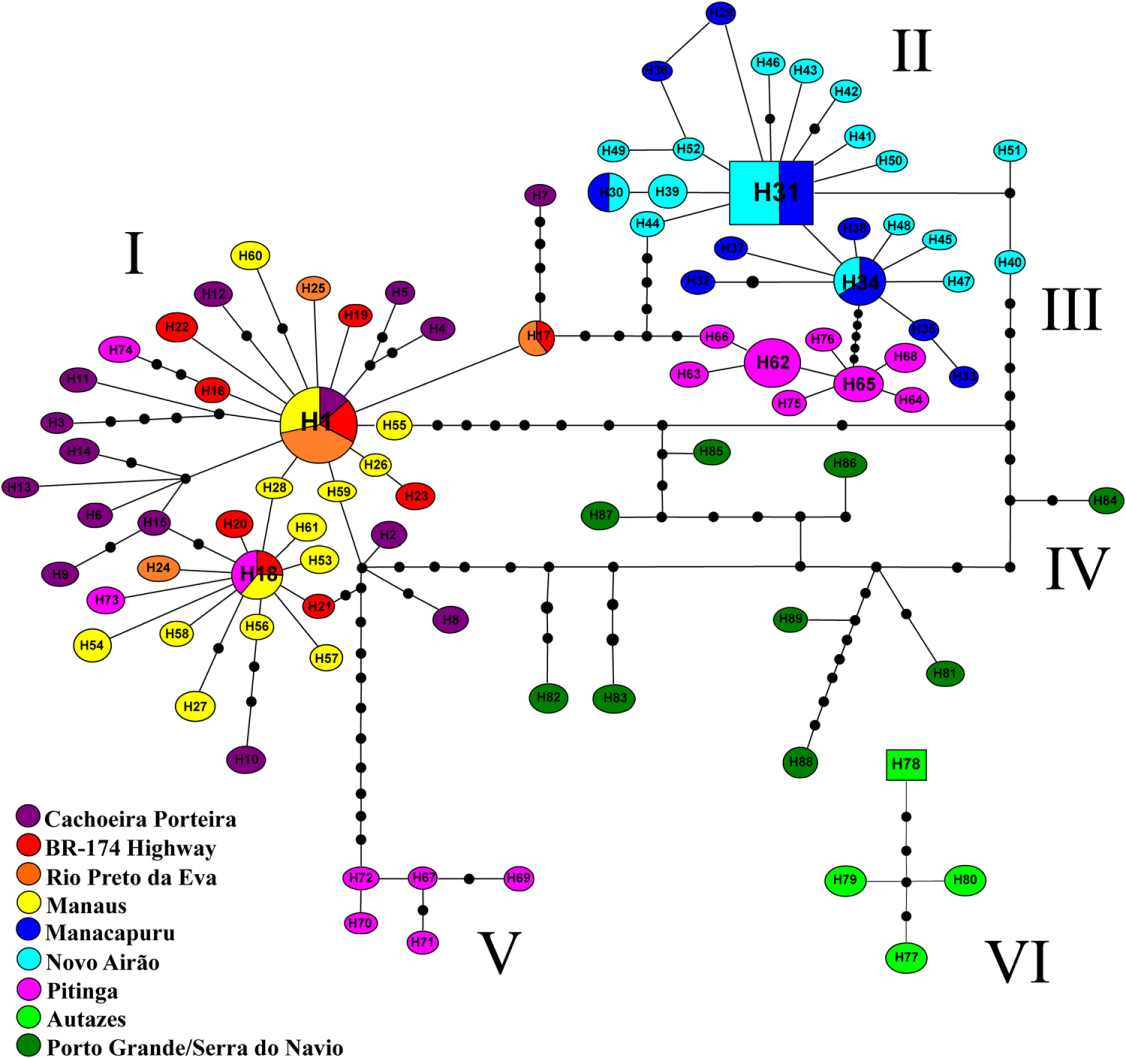
Parsimony haplotypes network of the 89 haplotypes obtained for *COI* in the nine *Lutzomyia umbratilis* populations from the Brazilian Amazon. H1 to H89 = haplotypes. I to VI = Haplogroups. The haplotype circle sizes are proportional to number of individuals observed in each haplotype (see Table S1). Full smaller circles (in black) represent mutational events.

For *COI*, the 176 individuals generated 89 haplotypes, of which 52 (H1 to H52) had been described by Scarpassa and Alencar^40^, and 37 being new haplotypes (H53 to H89) identified in this study. Of these, 79 (88.76%) haplotypes were singletons or exclusive to a single sample. Table S1 shows the haplotype frequencies in the samples studied. The Autazes and Porto Grande/Serra do Navio populations did not share haplotypes with any other locality. The haplotypes genealogy produced two disconnected networks and six haplogroups (Fig. 2). The larger network comprised five haplogroups (H1 to H76; H81 to H89). Haplogroup I clustered 48 haplotypes (H1 to H28; H53 to H61; H73, H74) corresponding to five localities from the north Amazon River and east Negro River: Cachoeira Porteira, BR-174 Highway, Rio Preto da Eva, Manaus and H18 (shared), H73, H74 from Pitinga. Haplogroup II clustered 24 haplotypes (H29 to H52), comprising two samples from north of the Amazon River and west of Negro River (interfluve 1): Manacapuru and Novo Airão. Haplogroup III clustered eight haplotypes (H62 to H66; H68; H75 and H76), corresponding to 24 individuals from Pitinga, north of the Amazon River and east of Negro River. Haplogroup IV clustered all haplotypes (H81 to H89) from the Porto Grande/Serra do Navio, north of the Amazon River and east of Negro River. Haplogroup V clustered five haplotypes (H67; H69 to H72), corresponding to five individuals from Pitinga, which were separated from the remaining haplotypes by at least 12 mutational steps. Haplogroup VI, disconnected from the remaining haplogroups, clustered four haplotypes (H77 to H80) from Autazes. This haplogroup differed from others by 13 fixed sites (eight transitions and five transversions). Here, we defined a fixed site as occurring when a mutation is present in all individuals from one population and absent in all individuals of the other populations.

**Figure 3:**
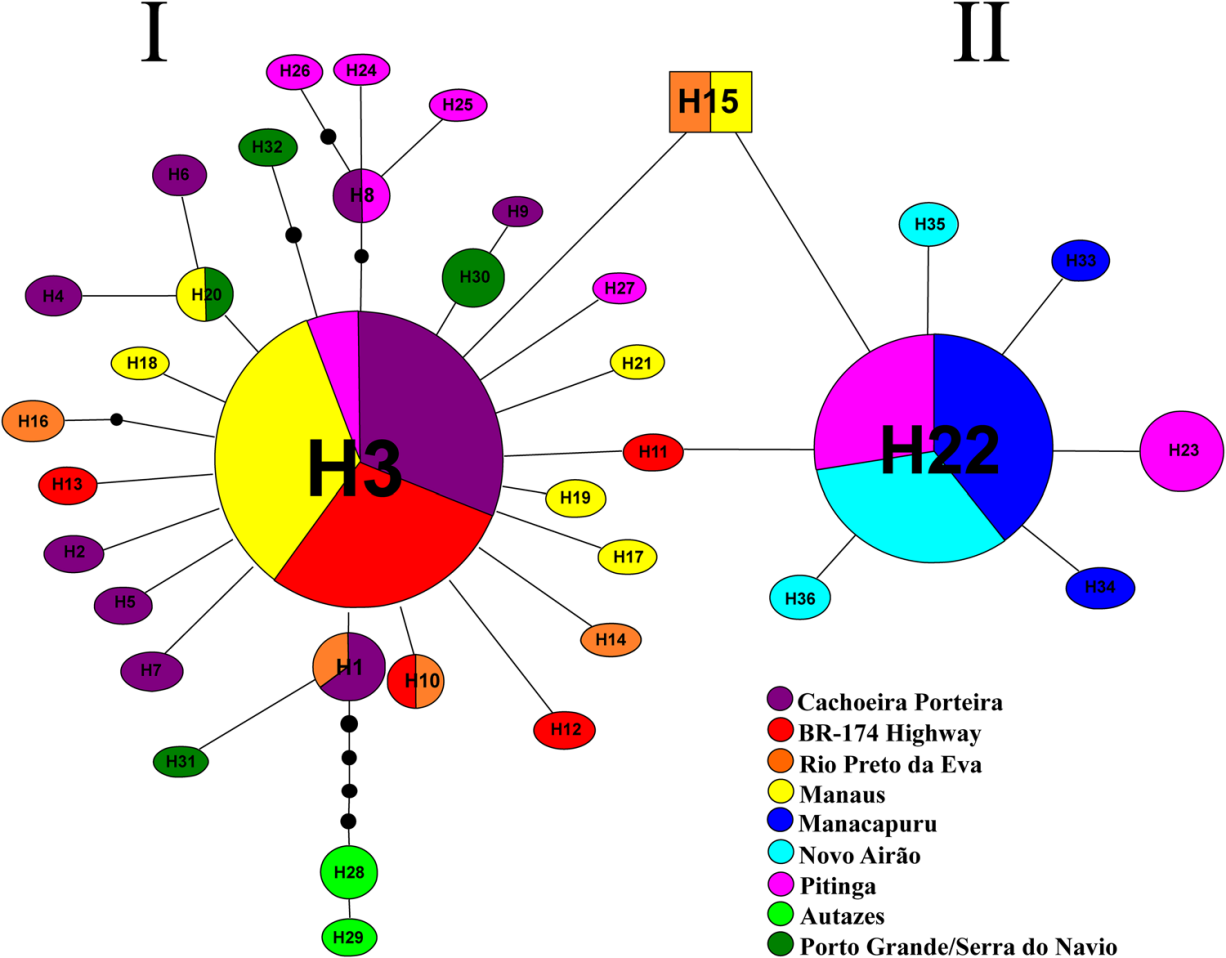
Parsimony haplotypes network of the 36 haplotypes obtained for *Cytb* in the nine *Lutzomyia umbratilis* populations from the Brazilian Amazon. H1 to H36 = haplotypes. I and II = Haplogroups. The haplotype circle sizes are proportional to number of individuals observed in each haplotype (see Table S1). Full smaller circles (in black) represent mutational events.

For *Cytb*, the 187 sequences generated 36 haplotypes, ranging from two (Autazes) to nine (Cachoeira Porteira) haplotypes per sample (Table S1; Fig. 3). Seven (19.44%) haplotypes were shared between localities and 29 (80.56%) were singletons or exclusive to a single sample. The Autazes sample did not share haplotypes with any other locality. All haplotypes were connected in a unique network; however, two star-shaped haplogroups (I and II) were observed; each one represented by a more frequent haplotype (H3 and H22, respectively), positioned more centrally in their respective haplogroups and connected by derived haplotypes, positioned peripherally. In haplogroup I, the H3 haplotype was shared among individuals all localities from north of the Amazon River (Fig. 3). The derived haplotypes from the Cachoeira Porteira, BR-174 Highway, Rio Preto da Eva, Manaus and Porto Grande/Serra do Navio and H8, H24 to H27 haplotypes from Pitinga were separated from H3 by one to four mutations. H28 and H29 haplotypes from Autazes were separated from H3 by seven and eight mutations, respectively.

In haplogroup II, H22 was the most common haplotype (Fig. 3). It was shared by 66 individuals situated on both sides of the Negro River (27 from Manacapuru, 22 from Novo Airão [both north of the Amazon River and west of Negro River, interfluve 1], and 17 from Pitinga [north of the Amazon River and east of Negro River]). H33 to H36 from Manacapuru and Novo Airão, and H23 from Pitinga were derived haplotypes and were connected to H22 by only one mutation. H11 (BR-174 Highway) and H15 (RP and MN) connected the two haplogroups by two mutations. In haplogroup I, H8 haplotype (derived) was shared by individuals of the Pitinga and Cachoeira Porteira (same bank) and could suggest contemporaneous gene flow, whereas in haplogroup II the sharing of H22 haplotype (likely the ancestral form) by individuals from the opposite banks could indicate historical genetic connectivity.

**Figure 4:**
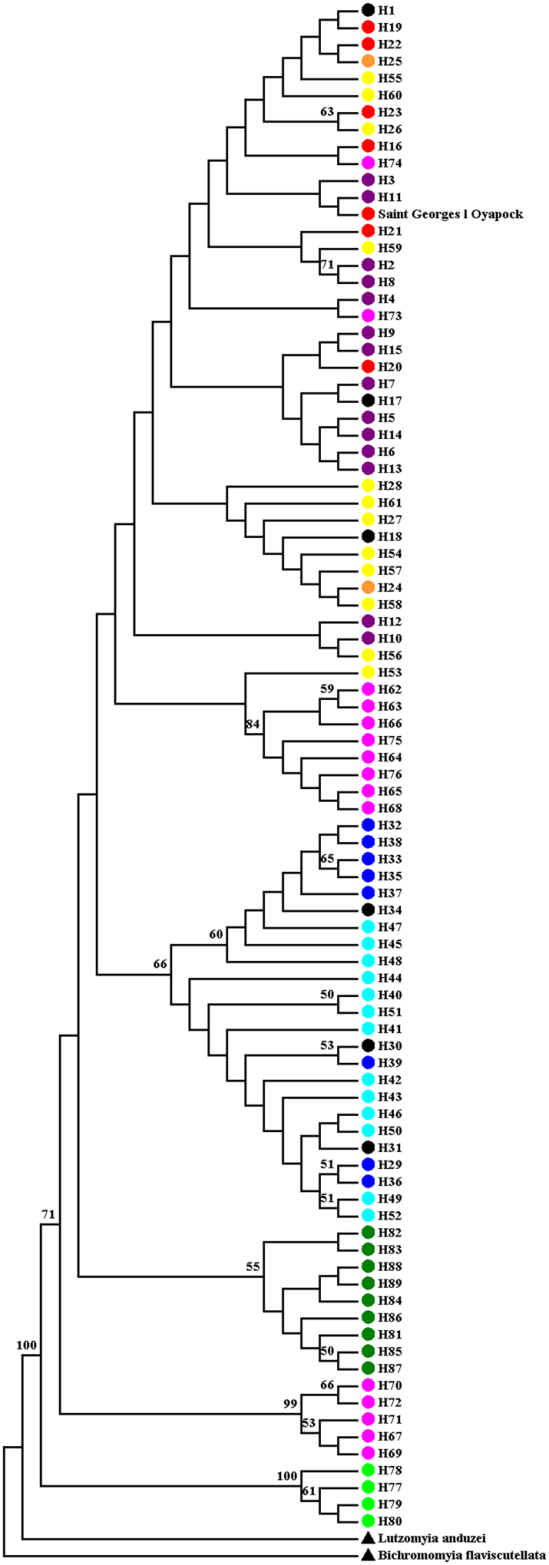
Neighbor-Joining (NJ) tree generated based on the 89 haplotypes of *COI.* Tree inferred under the Kimura 2-Parameters model and 5000 replications. The support values are indicated above of the branches. The colors on the terminal branches of the tree represent the haplotypes observed in each locality, following the same color pattern of the Figs. 1, 2 and 3. The black color on the terminal branches of tree represent the shared haplotypes between the localities. See Table S1 for identification of the haplotypes. Saint Georges l’ Oyapock, French Guiana. *Lutzomyia anduzei* and *Bichromomyia flaviscutellata* were used as outgroups.

Neighbor-Joining (NJ) tree topology inferred with the 89 haplotypes from *COI* retrieved five main groups (Fig. 4); however, most these showed weak support. Group 1, weakly supported, clustered the haplotypes from the Cachoeira Porteira (purple), BR-174 Highway (red), Rio Preto da Eva (orange), Manaus (yellow), the most haplotypes from Pitinga (pink) and the sequence of Saint Georges l’Oyapock, French Guiana. Group 2, moderately supported (66%), clustered all haplotypes from Manacapuru (dark blue) and Novo Airão (light blue). Group 3, weakly supported (55%), clustered all haplotypes from Porto Grande/Serra do Navio (dark green). Group 4, strongly supported (99%), clustered five haplotypes from Pitinga (pink). Group 5, the most basal and strongly supported (100%), clustered all haplotypes from Autazes (light green). Maximum Likelihood (ML) tree generated a very similar topology to Fig. 4 (Fig. S1).

**Figure 5:**
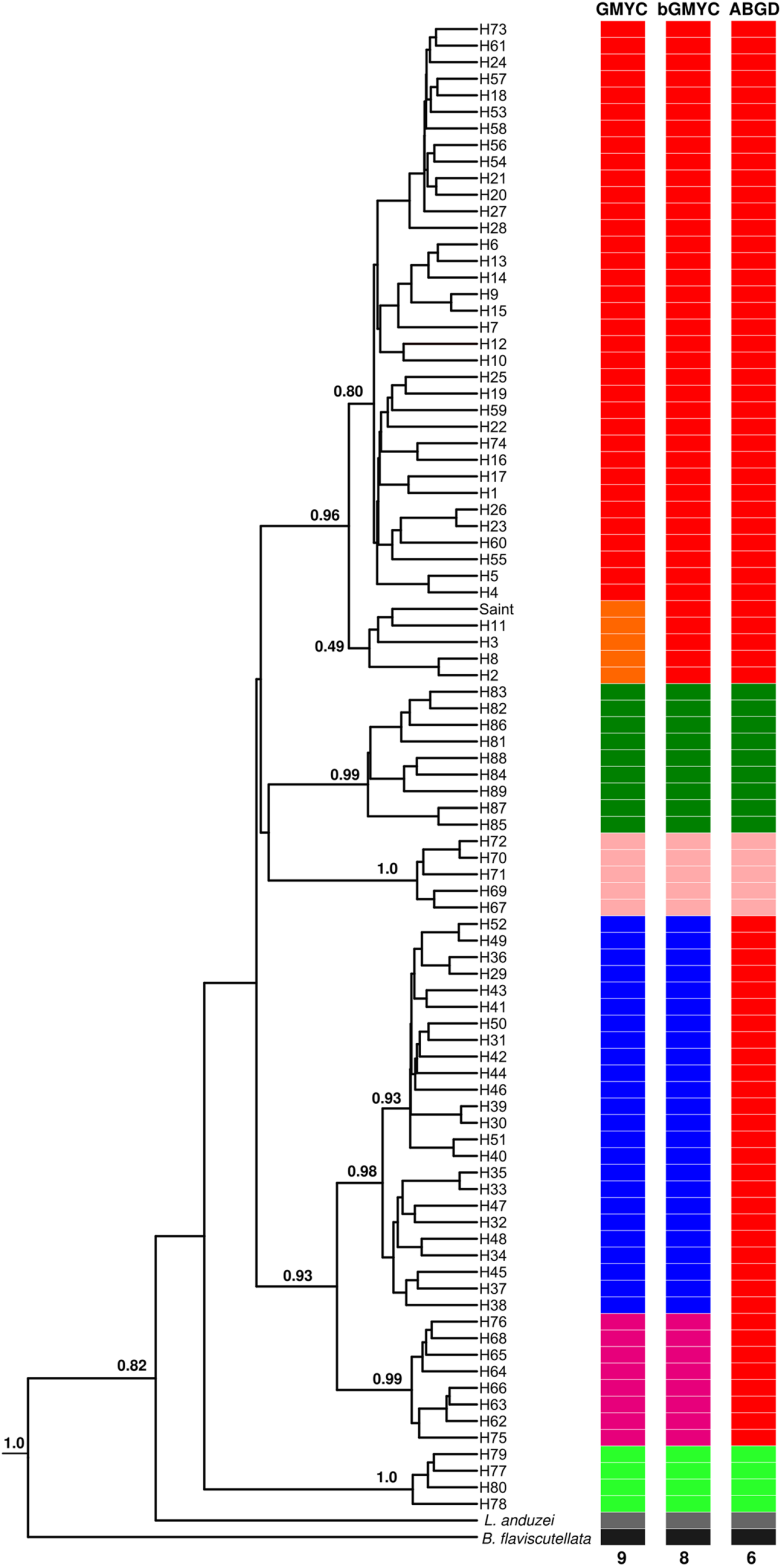
Bayesian Inference (BI) tree (maximum clade of credibility) based on the 89 haplotypes of *COI* and generated in BEAST. Tree inferred under the Time Reversible (GTR) + G + I nucleotide substitution model. The support values, in BPP (*Bayesian Posterior Probability*), are indicated above of the branches. See Table S1 for identification of the haplotypes. Saint: Saint Georges l' Oyapock, French Guiana. *Lutzomyia anduzei* and *Bichromomyia flaviscutellata* were used as outgroups. The species-genetic lineages delimited by the GMYC, bGMYC and ABGD models are represented by colored boxes. The 9, 8 and 6 indicate the lineage numbers recognized by the GMYC, bGMYC and ABGD models, respectively. Each color represents one lineage. The colors of the lineages delimitated by the three models followed the same pattern of the Fig. 2.

Bayesian Inference (BI) tree based on *COI* (Fig. 5) retrieved two major groups from *L. umbratilis*. The first group, the most basal, clustered all haplotypes from Autazes (BPP: 1.0). The second group consisted of two large subgroups: A) the most apical subgroup consisted three clusters: (A1) all haplotypes from the Cachoeira Porteira, BR-174 Highway, Rio Preto da Eva, Manaus plus H18 (shared), H73 and H74 from Pitinga and the Saint Georges l’Oyapock sequence (BPP: 0.96); (A2) all haplotypes from Porto Grande/Serra do Navio (BPP: 0.99); (A3) haplotypes (H67, H69-H72) from Pitinga, which formed sister cluster with Porto Grande/Serra do Navio (BPP: 1.0); (B) the most basal subgroup consisted two clusters (BPP: 0.93): (B1) all haplotypes from Manacapuru and Novo Airão (BPP: 0.98), and B2) haplotypes (H62-H66, H68, H75, H76) from Pitinga (BPP: 0.99), which formed sister cluster with Manacapuru and Novo Airão populations. In this analysis *L. anduzei* and *B. flaviscutellata* were used as outgroups.

The three GMYC, bGMYC and ABGD delimiters employed identified seven, six and four lineages for *L. umbratilis*, respectively (Fig. 5). The ABGD model was more conservative. In this model, the populations from north group (except Porto Grande/ Serra do Navio and lineage 5 from Pitinga) and Manacapuru and Novo Airão were included in the same lineage. However, the bGMYC model identified a lineages number (six) compatible with others analyses.

**Figure 6:**
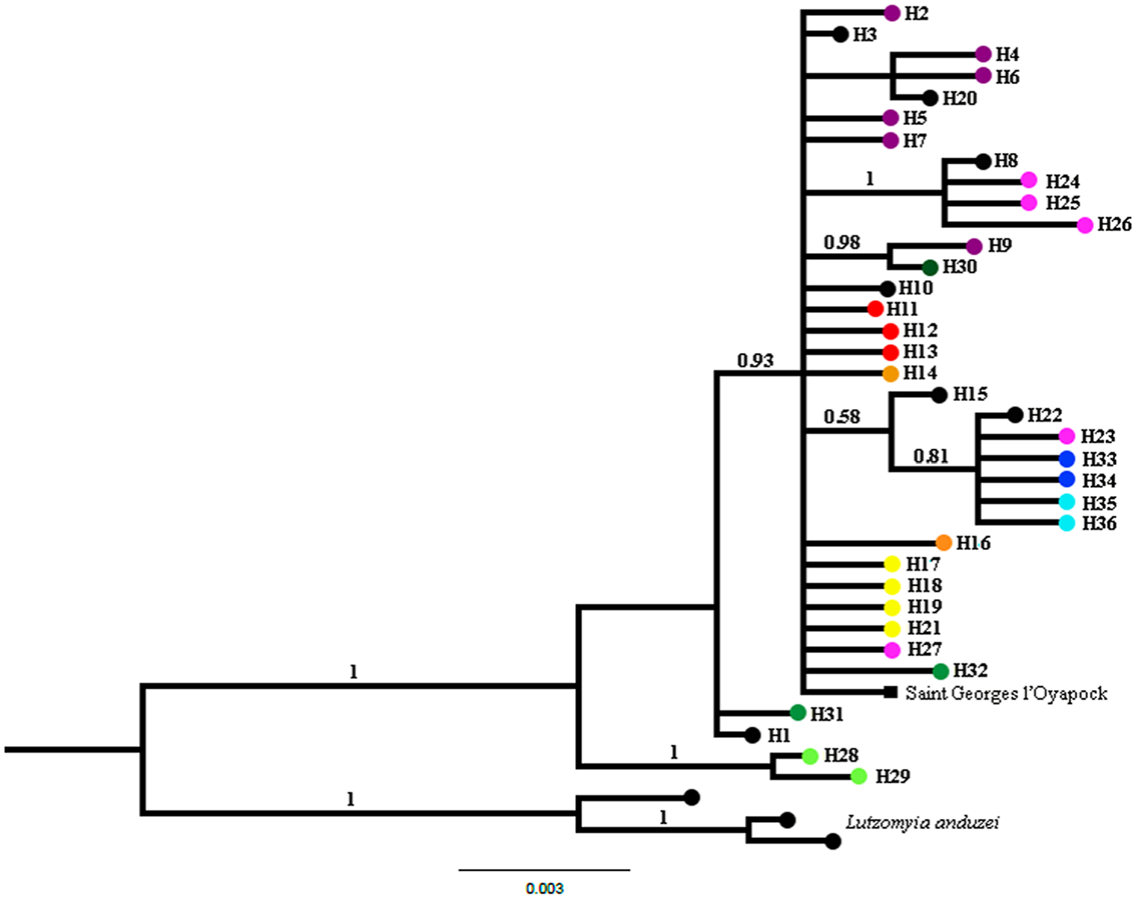
Bayesian Inference (BI) tree generated based on the 36 haplotypes of *Cytb*. Tree inferred under the HKY nucleotide substitution model. The support values, BPP (Bayesian Posterior Probability), are indicated above of the branches. The colors on the terminal branches of the tree represent the haplotypes observed in each locality, following the same color pattern of the Figs. 1, 2 and 3. The black color on the terminal branches of tree represent the shared haplotypes between the localities. See Table S1 for identification of the haplotypes. Saint Georges l’ Oyapock. French Guiana (square shape in black color). *Lutzomyia anduzei* was used as outgroup.

Neighbor-Joining (data not shown) and BI trees inferred with the 36 haplotypes from *Cytb* generated identical topologies. The groups were not well resolved, with polytomies and most branches having weak support. Figure 6 shows the BI tree topology. The most basal and the best resolved group was from Autazes (BPP: 1.0). The remaining haplotypes did not form defined groups. Therefore, it was not possible to establish evolutionary relationships between these groups. The absence of resolution may be due to the few informative sites (2.73%) recorded for this marker. In this analysis only *L. anduzei* was used as an outgroup due to the absence of *Cytb* sequences for *B. flaviscutellata*.

The BI tree generated in *BEAST v. 2.0^55^ (Fig. S2) shows the divergence times estimated between groups retrieved. The most recent cladogenesis event occurred between groups Cachoeira Porteira/BR-174 Highway/Rio Preto da Eva/Manaus/Pitinga (some haplotypes)/Porto Grande/Serra do Navio and Manacapuru/Novo Airão and was dated to ~ 180,000–230,000 years ago (Holocene). The node that indicates the oldest event occurred between Autazes and remaining groups and was dated to ~ 400,000–500,000 years ago (middle Pleistocene) (Fig. S2).

**Figure 7:**
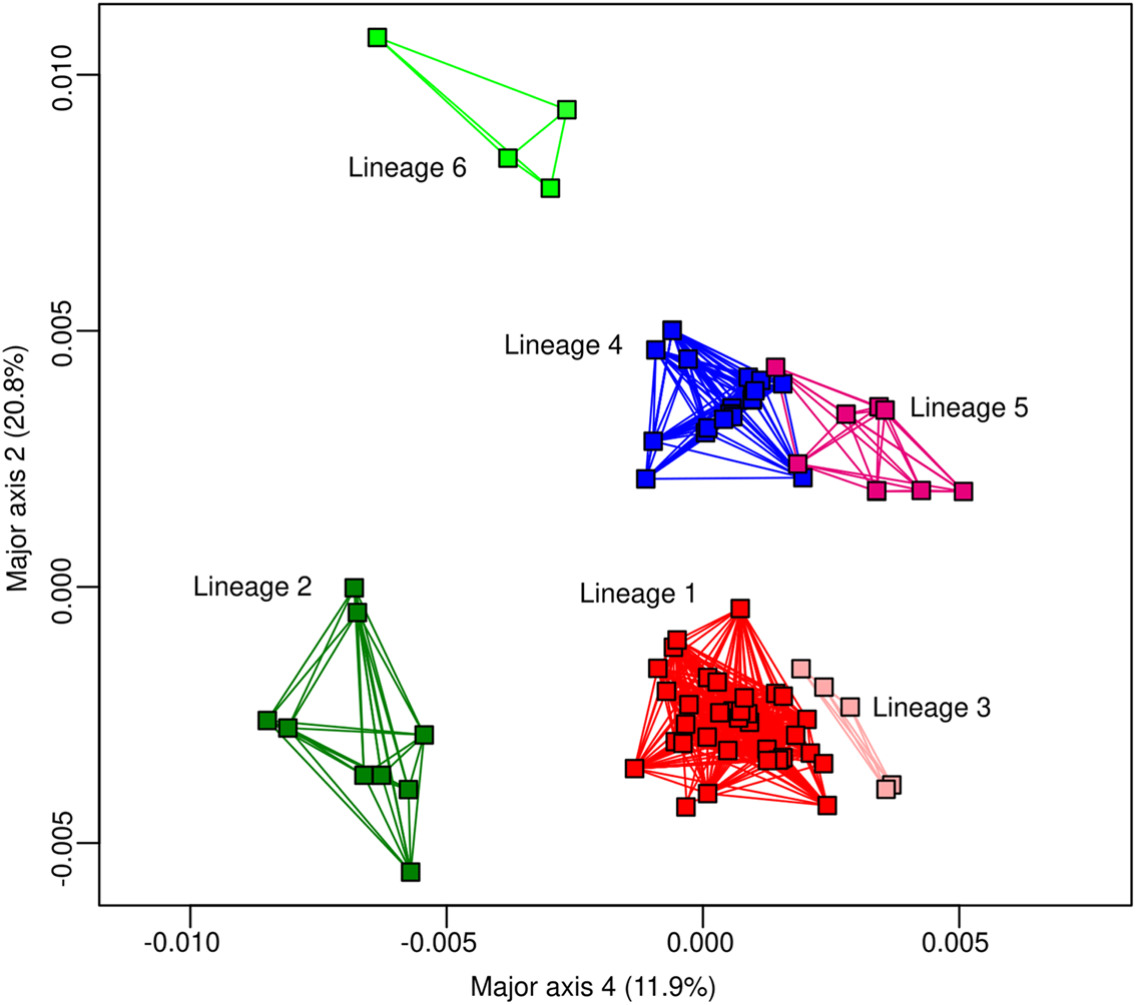
Principal Coordinates Analysis (PCA) generated based on the 89 haplotypes of *COI*. The colors of the lineages followed the same pattern of the Fig. 5.

PCA analysis clearly separated six genetic groups for the *COI* (Fig. 7), with the sample from Autazes (Lineage 6) being the most distant one.

Table S2 shows the intra-population genetic diversity measures and neutrality tests for *COI* and *Cytb*. Remarkably, genetic variation was higher in *COI* than *Cytb* for all samples, especially in the samples from Manacapuru and Novo Airão.

For *COI*, Tajima’s *D* test was negative, but not significant (*P* > 0.05), in the samples from Manaus, Pitinga, Autazes, Porto Grande/Serra do Navio (Table S2); therefore, the neutral model cannot be rejected. Fu’s *F*s test was negative and significant in samples from Manaus (*P* < 0.0001) and Porto Grande/Serra do Navio (*P* < 0.05), consistent with recent population expansion or positive selection. For *Cytb*, Tajima’s *D* and Fu’s *F*s tests were negatives and significant (*P* < 0.05) in samples from Cachoeira Porteira, BR-174 Highway, Rio Preto da Eva and Manaus, favoring the interpretation of recent population expansion or selective sweeps. This finding is compatible with the observed star-shaped haplotype network (Fig. 3).

Table S3 shows the pairwise genetic distances values, based on the *F*_ST_ statistic, among nine populations for the *COI* and *Cytb* genes. For *COI*, when the samples from Pitinga, Autazes and Porto Grande/Serra do Navio were integrated into the Scarpassa and Alencar dataset^40^, most pairwise comparisons had high and significant *F*_ST_ values, regardless whether the compared populations were situated on the same or opposite sides of the rivers and between interfluves. This result is due to the genetic subdivision of the Pitinga sample, and the highly divergent haplotypes of Porto Grande/Serra do Navio. Nonetheless, the highest and most significant *F*_ST_ values were observed in the comparisons involving the Autazes sample, that ranged from 0.7171 (Pitinga *versus* Autazes) to 0.9333 (Rio Preto da Eva *versus* Autazes).

For *Cytb*, the results obtained for the six samples (Cachoeira Porteira, BR-174 Highway, Rio Preto da Eva, Manaus, Manacapuru and Novo Airão) were similar those described for *COI* by Scarpassa and Alencar^40^, where low genetic distances were observed between localities situated on the same side of the rivers and within interfluves, whereas high and significant genetic distance values were detected between localities from opposite sides and between interfluves. But, when the Pitinga, Autazes and Porto Grande/Serra do Navio samples were included in the comparisons, the genetic distance values varied depending on the sample. Higher *F*_ST_ values were observed (0.4870) between Manaus *versus* Pitinga (same side) than between Manacapuru *versus* Pitinga samples (0.1559) and Novo Airão *versus* Pitinga (0.1395), situated on opposite sides of the rivers.

Table S3 also provides the average number of nucleotide substitutions per site between samples (*D*_xy_), the number of net nucleotide substitutions per site between samples (*D*_a_), the number of shared polymorphisms between pairs of samples (*S*_s_), and the number of fixed differences between pairs of samples (*S*_f_) for the two genes. For *COI*, similarly *F*_ST_ statistic, the highest *D*_xy_, *D*_a_ and *S*_f_ values were found in the comparisons between Autazes *versus* Cachoeira Porteira and between Autazes *versus* Manacapuru (both with *S*_f_ = 15), and between Autazes *versus* Manaus and between Autazes *versus* Porto Grande/Serra do Navio (both with *S*_f_ = 18). For *Cytb*, the highest values were between Autazes *versus* Manacapuru and between Autazes *versus* Novo Airão (both with *S*_f_ = 8).

As in the previous analyses, the BAPS analyses identified six genetic groups for *COI*, and three for *Cytb* (log ML = −2028.6632 and −564.0626; posterior probability = 0.9999 and 0.9784, respectively) (Figs. S3 and S4).

Genetic distance (uncorrected-*p* distance) was also calculated between lineages retrieved from the haplotype networks (Figs. 2 and 3). Table 2 shows *COI* genetic distances. These ranged from 0.7% (between lineages I and III and between lineages II and III) to 2.3% (between lineages IV and VI). Between lineages and *L. anduzei* and *B. flaviscutellata* s.s. the mean genetic distances were 5.7% and 11.9%, respectively. Table 3 gives the *Cytb* genetic distances. These ranged from 0.05% (between lineages I and II) to 1.7% (between lineages II and III). Between the three lineages and *L. anduzei* genetic distances varied from 4.4% to 4.5%.

**Table 2:** Mean values of genetic distance (uncorrected-*p* distance) and their respective standard errors (mean ± SE) estimated among the *L. umbratilis* lineages (haplogroups) and the outgroups, based on the *COI* gene.

Lineages	I	II	III	IV	V	VI	L. anduzei*	B. flaviscutellata*
I	**0.3% ± 0.001**							
II	0.9% ± 0.002	**0.1% ± 0.000**						
III	0.7% ± 0.002	0.7% ± 0.002	**0.1% ± 0.000**					
IV	1.1% ± 0.003	1.1% ± 0.003	1.3% ± 0.003	**0.7% ± 0.001**				
V	1.3% ± 0.004	1.4% ± 0.004	1.5% ± 0.004	1.6% ± 0.004	**0.2% ± 0.001**			
VI	1.8% ± 0.002	2.1% ± 0.002	1.9% ± 0.003	2.3% ± 0.003	2.2% ± 0.004	**0.3% ± 0.001**		
L. anduzei*	5.6% ± 0.006	5.5% ± 0.006	5.5% ± 0.006	5.8% ± 0.006	5.8% ± 0.006	5.8% ± 0.006	**0.5% ± 0.001**	
B.flaviscutellata*	11.9% ± 0.009	12.0% ± 0.009	11.8% ± 0.009	11.8% ± 0.009	11.8% ± 0.009	12.2% ± 0.009	11.7 ± 0.009	**0.5% ± 0.001**

For localization of the lineages (haplogroups), see Fig. 2. The values in **bold** in the diagonal represent the intraspecific distances. Mean distance in the ingroup = 1.6% ± 0.002. * = outgroups. Haplogroup I corresponds Lineage 1 (red) of Fig. 5; Haplogroup II corresponds Lineage 4 (blue) of Fig. 5; Haplogroup III corresponds Lineage 5 (pink) of Fig. 5; Haplogroup IV corresponds Lineage 2 (dark green) of Fig. 5; Haplogroup V corresponds Lineage 3 (light pink) of Fig. 5; Haplogroup VI corresponds Lineage 6 (light green) of Fig. 5 (bGMYC model).

**Table 3:** Mean values of genetic distance (uncorrected-*p* distance) and their respective standard errors (mean ± SE) among the *L. umbratilis* lineages (haplogroups) from the Brazilian Amazon and the outgroup, based on the *Cytb* gene.

Lineages	I	II	III	L. anduzei*
I	**0.02% ± 0.000**			
II	0.05% ± 0.003	**0.01% ± 0.000**		
III	1.3 ± 0.005	1.7% ± 0.006	**0.01% ± 0.001**	
L. anduzei*	4.4% ± 0.009	4.4% ± 0.009	4.5% ± 0.010	**0.06% ± 0.003**

For localization of the lineages (haplogroups), see Fig. 3. The values in **bold** in the diagonal represent the intraspecific distances. Mean distance in the ingroup = 1.02 ± 0.003. * = outgroup.

A Mantel test was used to analyze the correlation between genetic and geographic distances among samples for each marker. These analyses showed that there was no correlation between genetic and geographic distances for either marker (*COI*: *r* = 0.128849, *P* = 0.2730; *Cytb*:* r* = -0.110475, *P* = 0.4825).

## Discussion

Population genetics through access to population structure analyses, demographic history and evolutionary relationships has become an indispensable discipline in evolutionary biology, allowing the discovery of evolutionary lineages and hidden species.

The findings of this study on population structure (Figs. 2, 3; Table S3) revealed a deep and significant genetic split for *L. umbratilis* from the Brazilian Amazon, confirming the previous biological and molecular studies^33,35,38,40,41^. On the other hand, phylogenetic analyses with *COI* identified up to six groups, but only the groups represented by five (H67; H69 to H72) haplotypes from Pitinga (haplogroup V; Fig. 2) and the sample from Autazes were strongly supported in most of the analyses (Figs. 4, 5). For *Cytb*, the BAPs analysis indicated three genetic groups, whereas in the phylogenetic analyses, the tree topologies showed little resolution preventing to establish the evolutionary relationships among the groups, except for the Autazes population, that as was found in *COI*, formed a group strongly supported (Fig. 6). Thus, the weak support observed for most groups and the absence of reciprocally monophyletic clades suggest an incomplete lineage sorting caused by retention of ancestral polymorphism due to the recent evolutionary timing of the *L. umbratilis* diversification process^72^.

Populations situated north of the Amazon River and east of Negro River (Cachoeira Porteira, BR-174 Highway, Rio Preto da Eva, Manaus, H18, H74 and H75 haplotypes of Pitinga) showed shared haplotypes and low genetic differentiation, with no or few fixed sites, and having most haplotypes clustered within the same haplogroup, as well as in phylogenetic analyses (Figs. 2, 3 and 5; Table S3) reflecting ongoing gene flow or a recent common history for these populations. This outcome indicates a degree of structuring consistent with populations within a single species^73^, consequently, they represent the same lineage (Lineage I; Fig. 1B). Nonetheless, the sample of Porto Grande/Serra do Navio, also situated north of the Amazon River and east of Negro River, revealed some degree of genetic structure (Figs. 2, 5; Table S3) and it was recognized as a distinct lineage with three models of delimitation used (Fig. 5), as well as in the PCA analysis (Fig. 7). However, the BAPS results for *Cytb* (Fig. S4) identified this population genetically similar to those of the group northern as well as the results of genetic structure (Table S3) provided no evidence for historic isolation between this and the remaining populations from group north. Thus, it also was included in the Lineage I (Fig. 1b). It is possible that additional analysis of the Porto Grande/Serra do Navio population using a larger sample size, would reduce these differences. Based on the phylogenetic analyses, *L. umbratilis* from Saint-Georges l’Oyapock^48^ also belongs to the same lineage, as does the population from the state of Pernambuco^41^.

Like *COI*^40^, the *Cytb* analyses also found that populations from Manacapuru and Novo Airão, situated in the interfluve of the north Amazon River and west of Negro River, were genetically similar to each one (Figs. 3, 6; Table S3); accordingly, they represent the same lineage or species. On the other hand, well-marked genetic differences, with *N*m values < 1, absence of shared sites and presence of fixed sites, were observed between populations from Manacapuru/Novo Airão and those from areas north (except Pitinga) of the Amazon River and east of Negro River (*S*_s_ = 0 and *S*_f_ = 6–7), and between Manacapuru/Novo Airão and Autazes (**interfluves 1** *versus* **2**; *S*_s_ = 0 and *S*_f_ = 8). These findings reinforce the evidences that the Manacapuru and Novo Airão populations could represent a distinct evolutionary lineage (Lineage II; Fig. 1b), although the genetic differentiation was low (from 0.7% to 2.1%; Table 2) and for the *Cytb,* they shared haplotypes with the Pitinga population (Fig. 3). The differences in levels of diversity and genetic structure between COI and Cytb genes observed here indicate that they evolve at different rates, with Cytb seeming to evolve more slowly, consequently it shows an older evolutionary history for these populations compared with the highly variable COI gene.

The specimens from Autazes generated *COI* haplotypes very different (from 13 to 18 fixed sites) from the remaining specimens studied here. This was shown by a disconnected haplotype network (Fig. 2), the highest genetic distance values and an absence of shared sites (*S*_s_) in the most comparisons (Tables 2 and S3), clearly indicating the genetic discontinuity of this population. In the phylogenetic analyses with both markers, haplotypes from Autazes were clustered in a strongly supported basal group, demonstrating an independent evolutionary trajectory for this lineage. This evidence, allied to a large number of fixed sites (Table S3), suggests historic fragmentation between the Autazes population and the other populations analyzed, and indicates that its divergence process may have started earlier. The municipality of Autazes is situated in the interfluve between Amazon and Madeira Rivers (named here interfluve 2). Taken together, the Amazon River may act as significant barrier, preventing the gene flow between Autazes populations and the remaining populations from the opposite banks, so promoting genetic differentiation. Based on this evidence, we suggested this population, first reported in this study, could represent a new lineage within *L. umbratilis* (Lineage III; Fig. 1b).

The IBD-based hypothesis was rejected for both markers, suggesting that most of the genetic subdivision observed in *L. umbratilis* populations cannot be explained by geographic distance, without physical barrier. As previously observed^40^, when sandfly populations from the same side of the rivers and those within interfluves are compared, they exhibited very low genetic differentiation, even when separated by large geographic distances, that may range from ~ 354 to 369 km (Table S3). Otherwise, when comparisons are made between populations from the opposite sides of rivers and between interfluves, very high and significant genetic differentiation were observed, even when these populations are separated by short geographical distances, ranging from ~ 59 to 96 km^10^. However, the exceptions were observed between Pitinga and the remaining locations from northern Amazon and east of Negro Rivers and between Pitinga and Manacapuru/Novo Airão.

In recent decades, the Amazon River and its tributaries have been the focus of many phylogeographical and biogeographical studies on their potential role in population isolation^10,11,74–85^. Studies have suggested that this complex river system most likely originated in the Miocene, ~ 11 Mya, and took its present shape in the late Pliocene, ~ 2.4 Mya^86^. Rossetti and others^87^ proposed that the Amazon River reached its current flow during the late Pleistocene. In this study, the separation time estimated between groups falls from middle Pleistocene to Holocene (Fig. S2), coinciding with the drainage system of Amazon River^86,88^. In this context, our findings provide support for the proposal that the Amazon and Negro Rivers and their tributaries are the main evolutionary forces driving the diversification process by allopatry in *L. umbratilis*^40^. Besides this vicariant event, the low dispersal rate of these sandflies, which seldom move more than 1 km from their birth place^6,7^, along with the amenable environmental conditions for adaptation and also drift may have contributed to the emergence and marked differentiation of the three main genetic lineages observed in this study.

Curiously, molecular studies conducted with other sandflies from the Brazilian Amazon, such as *L. anduzei*^5^, *B. flaviscutellata* and *Bichromomyia nociva*^89,90^, which we presumed to have similar dispersion patterns, did not support this fluvial system acting as physical barriers between populations of these species. Such differences may suggest either lack of a common evolutionary history affecting these species or distinct dispersion rates for these species. Further studies will be required to support or refute these hypotheses.

### Two or three species?

Lineage delimitation based on the bGMYC model identified six lineages for *L. umbratilis* of this study (Fig. 5), a result compatible with the haplotype network, phylogenetic analyses, BAPS and PCA analyses for *COI.* For both markers, the haplotypes from Pitinga population segregated into different haplogroups in the networks (Figs. 2, 3) and in the phylogenetic analyses (Figs. 4, 5, 6). Two (GMYC and bGMYC) of the three models used recognized three distinct lineages within Pitinga (Fig. 5) indicating admixture or an expressive genetic subdivision for this population. Based on this, could the Pitinga population be a polytypic lineage or species? PCA analysis (Fig. 7) indicated that the Lineage 3 from Pitinga is more closely related to remaining populations from group north (Lineage 1), as well as the genetic distance was low between them (1.3%; Table 2), supporting close relationship. PCA analysis (Fig. 7) also showed that the Lineage 5 from Pitinga is more genetically closely related to Lineage 4 from Manacapuru and Novo Airão (0.7%; Table 2) and for the *Cytb*, the Pitinga population shared haplotypes with the Manacapuru and Novo Airão populations. It is possible that in recent past the individuals from Pitinga had contact with individuals from Novo Airão and/or neighboring areas, via the Anavilhanas archipelago situated on Negro River (Fig. 1a). Anavilhanas archipelago is second largest group of freshwater islands in the world and comprises an intricate pattern of islands, channels, lagoons, swamps, and partially submerged sandbanks that are refuge for a diversified fauna and flora. This archipelago is located on the lower course of the Negro River, where the widest part of the river may reach approximately 20 km^91^. Thus, the dispersal of the individuals would have occurred step by step across of these islands, especially in the dry season when the land span of the islands may be greater. Hence, this region from Negro River would be acting (or acted) as a porous barrier to gene flow between populations of the opposite sides in this stretch of River. Further investigations, focusing on a broader sampling and the use of genomic approaches, such as RADseq that provide higher resolution to detect genetic structure and heterozygous individuals, will be needed to clarify these findings recorded at Pitinga.

On the other hand, the Lineage 6 (Autazes) was the most isolated from remaining lineages (Fig. 7); a result congruent with haplotype network, genetic structure and phylogenetic analyses. The population of Autazes is situated to south Amazon River and did not share haplotypes with any other population analyzed. Amazon River is considered the widest in the world and in the rainy season it may reach up ~ 50 km wide. Thus, the genetic isolation observed for Autazes population indicates that, unlike the Negro River, the Amazon River acts as effective barrier to the dispersal between individuals from opposites banks, as consequence the Autazes population was the most divergent.

The values for uncorrected-*p* distances were low between lineages (*COI*: from 0.7 to 2.3%; *Cytb*: from 0.05% to 1.7%), contrasting with very high *F*_ST_ values between samples (Table S3). Studies carried out with other Diptera have also reported low genetic distance values between cryptic species, such as *Anopheles albitarsis* (1.7–3.5%)^92^, *Anopheles triannulatus* (1.7–2.3%)^93^ and *Anopheles nuneztovari* (1.6–2.8%)^94^ complexes, which characterize these species as of recent evolutionary origin. Thus, the differentiation level observed here is indicative of recent divergence between lineages, supporting our conclusions as reported above. This occurs due to the fact that, in young species, fixed differences may be observed only in genes involved in the speciation process^95^.

Previous studies conducted with *L. umbratilis* populations north of Amazon/east of Negro Rivers and north of the Amazon/west of Negro Rivers revealed appreciable and significant biological differences between them^38^. Justiniano and others^38^ compared the first laboratory bred generations of *L. umbratilis* populations obtained of the opposite banks of the Negro River (Manaus and Manacapuru). Interestingly, these samples showed remarkable differences in their life cycle, fecundity, fertility and adult longevity, with the population from Manaus being more productive and living longer than the population from Manacapuru. Such differences could be due to intrinsic biological features resulting to distinct evolutionary adaptations due to their geographical isolation by the Negro River. On the other hand, Justiniano^39^ observed discrete morphological differences in the immature stage, and in the number and size of the spines of armature of the female genital atrium between populations of these locations. The results of this study support the previous biological^38^ and molecular studies^33,35,40,41^ and, when taken together, it is possible to infer that the populations north and south of the Amazon River and interfluves represent distinct “species”, which could have diverged more biological and molecular levels than morphologic^39^ level, with implications distinct epidemiologic^13,37^. Genomics approach using loci involved in the different biologic aspects of *L. umbratilis* may clarify this issue. For example, some of these efforts may be focused on the mechanisms that determine the efficiency as vector, as well as to identify specific loci (outliers) that are acting in the most variable adaptability processes of these lineages. In this study, we also recorded a new independently evolving lineage in Autazes. Hence, our findings provide convincing arguments in favor of the hypothesis that what is currently termed *L. umbratilis* represents, in fact, at least three evolutionary lineages, possibly species, in the studied area.

Overall, the first lineage occurs north of the Amazon and east of Negro Rivers (Lineage I; Fig. 1b), where *Le. guyanensis* transmission is intense, implying that *L. umbratilis* is an important vector there. The second lineage occurs in the interfluve between north of the Amazon and west of the Negro Rivers (Lineage II; Fig. 1b), previously reported to be an area free of *Le. guyanensis* transmission. The third lineage, first recorded by this study, occurs in the interfluve between south of the Amazon and west of the Madeira Rivers (Lineage III; Fig. 1b) and was the most divergent lineage and could be the most ancestral. The involvement of this lineage in *Leishmania* transmission remains to be elucidated and should be investigated further. The first lineage is most likely the type form (*L. umbratilis s.s.*), because it occurs in the same bank as the type locality (Monte Dourado area of the Jari River, state of Pará, north of the Amazon and east of the Negro Rivers)^31^, so that the other two taxa therefore represent unrecognized species.

In conclusion, our findings revealed *L. umbratilis* three lineages in the Brazilian Amazon. The origin of these lineages is most likely to be associated with the formation of the Amazon drainage system and its tributaries. The first and second lineages are on the north of the Amazon/east of the Negro Rivers and north of the Amazon/west of the Negro Rivers, respectively; the third lineage is known from the interfluve between south of the Amazon River and west of the Madeira River. The three lineages may represent three species. However, their confirmation will require detailed examination morphological of immature and adult stage combined with molecular studies to provide diagnostic characters that can allow them to be recognized as valid species. Given the strong evidence of genetic subdivision within the Pitinga population, we do not rule out the possibility that more lineages or species exist in the Brazilian Amazon, as well as in other locations unsampled in northern South America. These findings will aid epidemiological studies, surveillance and vector control programs in these regions, especially north of the Amazon River, where the transmission by *Le. guyanensis* can be very high.

## Supplementary Information

Supplementary Figure S1.

Supplementary Figure S2.

Supplementary Figure S3.

Supplementary Figure S4.

Supplementary Information.

Supplementary Table S1.

Supplementary Table S2.

Supplementary Table S3.
