# Publisher Correction: Multiple evolutionary lineages for the main vector of *Leishmania guyanensis, Lutzomyia umbratilis* (Diptera: Psychodidae), in the Brazilian Amazon

**DOI:** 10.1038/s41598-021-96387-4

**Published:** 2021-08-19

**Authors:** Vera Margarete Scarpassa, Antônio Saulo Cunha-Machado, Ronildo Baiatone Alencar

**Affiliations:** 1 Laboratório de Genética de Populações e Evolução de Mosquitos Vetores, Coordenação de Biodiversidade, Instituto Nacional de Pesquisas da Amazônia, Avenida André Araújo, 2936. Bairro Aleixo, Manaus, Amazonas CEP 69.067-375 Brazil; 2 Laboratório de Biologia Molecular, Centro de Biotecnologia da Amazônia, Avenida Governador Danilo Areosa s/n. Distrito Industrial I, Manaus, Amazonas CEP 69.075-351 Brazil; 3 Departamento de Vigilância Epidemiológica, Fundação de Vigilância em Saúde do Estado do Amazonas, Manaus, Amazonas CEP 69.093-018 Brazil

Correction to: *Scientific Reports* 10.1038/s41598-021-93072-4, published online 28 July 2021

The original version of this Article contained errors.

In the Abstract,

“The third lineage, first recorded in this study, is in the interfluve between south of Amazonas River and west of Madeira River, and its involvement in the transmission of this parasite remains to be elucidated.”

now reads:

“The third lineage, first recorded in this study, is in the interfluve between south of Amazon River and west of Madeira River, and its involvement in the transmission of this parasite remains to be elucidated.”

In addition, a previous rendition of Figure 5 was published. As a result, the colored boxes were incorrectly shown.

The original Figure 5 and accompanying legend appear below.

**Figure 5:**
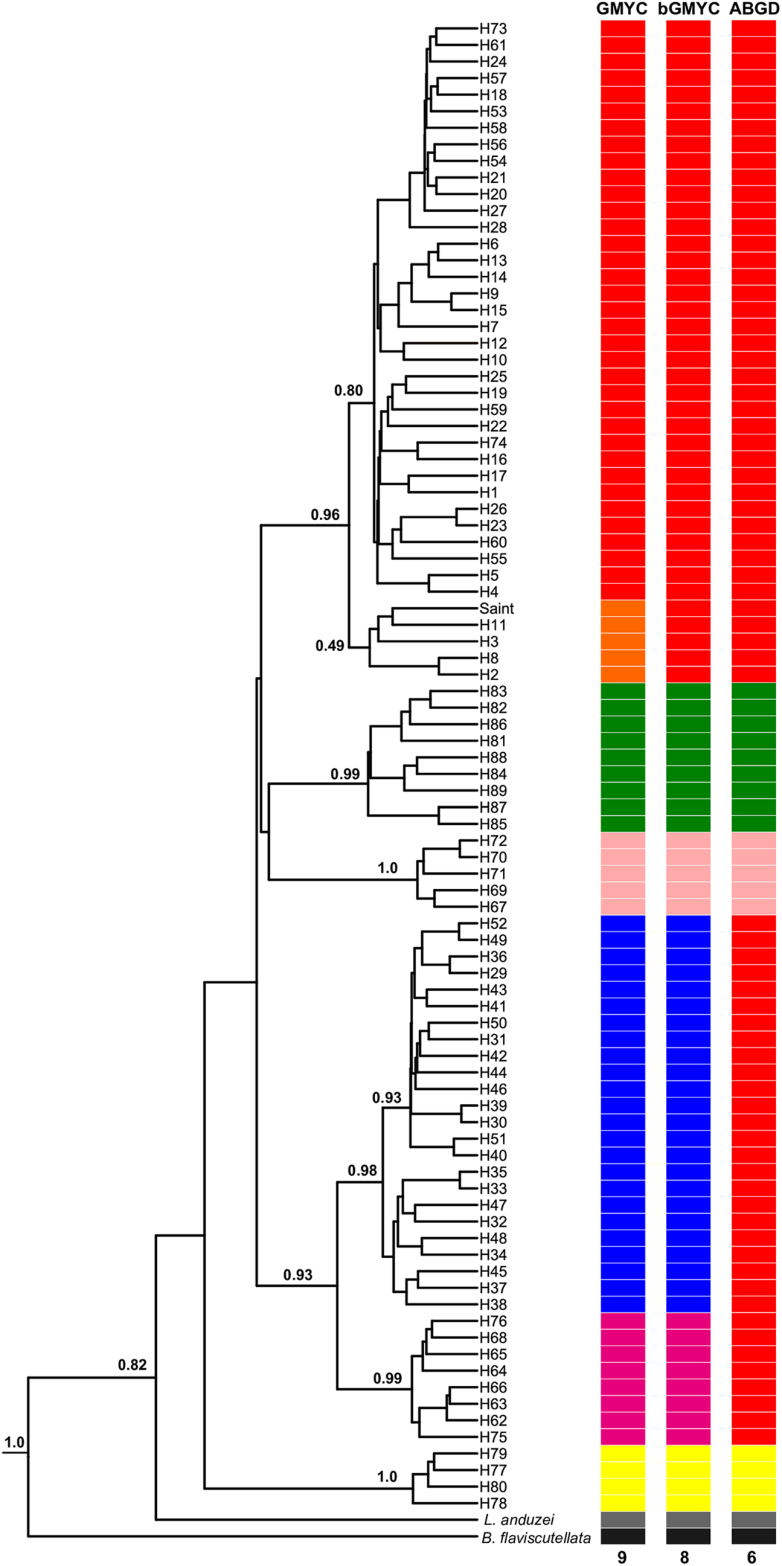
Bayesian Inference (BI) tree (maximum clade of credibility) based on the 89 haplotypes of *COI* and generated in BEAST. Tree inferred under the Time Reversible (GTR) + G + I nucleotide substitution model. The support values, in BPP (Bayesian Posterior Probability), are indicated above of the branches. See Table S1 for identification of the haplotypes. Saint: Saint Georges l’ Oyapock, French Guiana. *Lutzomyia anduzei* and *Bichromomyia flaviscutellata* were used as outgroups. The species-genetic lineages delimited by the GMYC, bGMYC and ABGD models are represented by colored boxes. The 9, 8 and 6 indicate the lineage numbers recognized by the GMYC, bGMYC and ABGD models, respectively. Each color represents one lineage. The colors of the lineages delimitated by the three models followed the same pattern of the Fig. 2.

The original Article has been corrected.

