# Supplementary figures and images for: Multiple evolutionary lineages for the main vector of Leishmania guyanensis, Lutzomyia umbratilis (Diptera: Psychodidae), in the Brazilian Amazon
Source: Sci Rep. 2021 Jul 28;11:15323. doi: 10.1038/s41598-021-93072-4 (PMC8319306; doi:10.1038/s41598-021-93072-4)

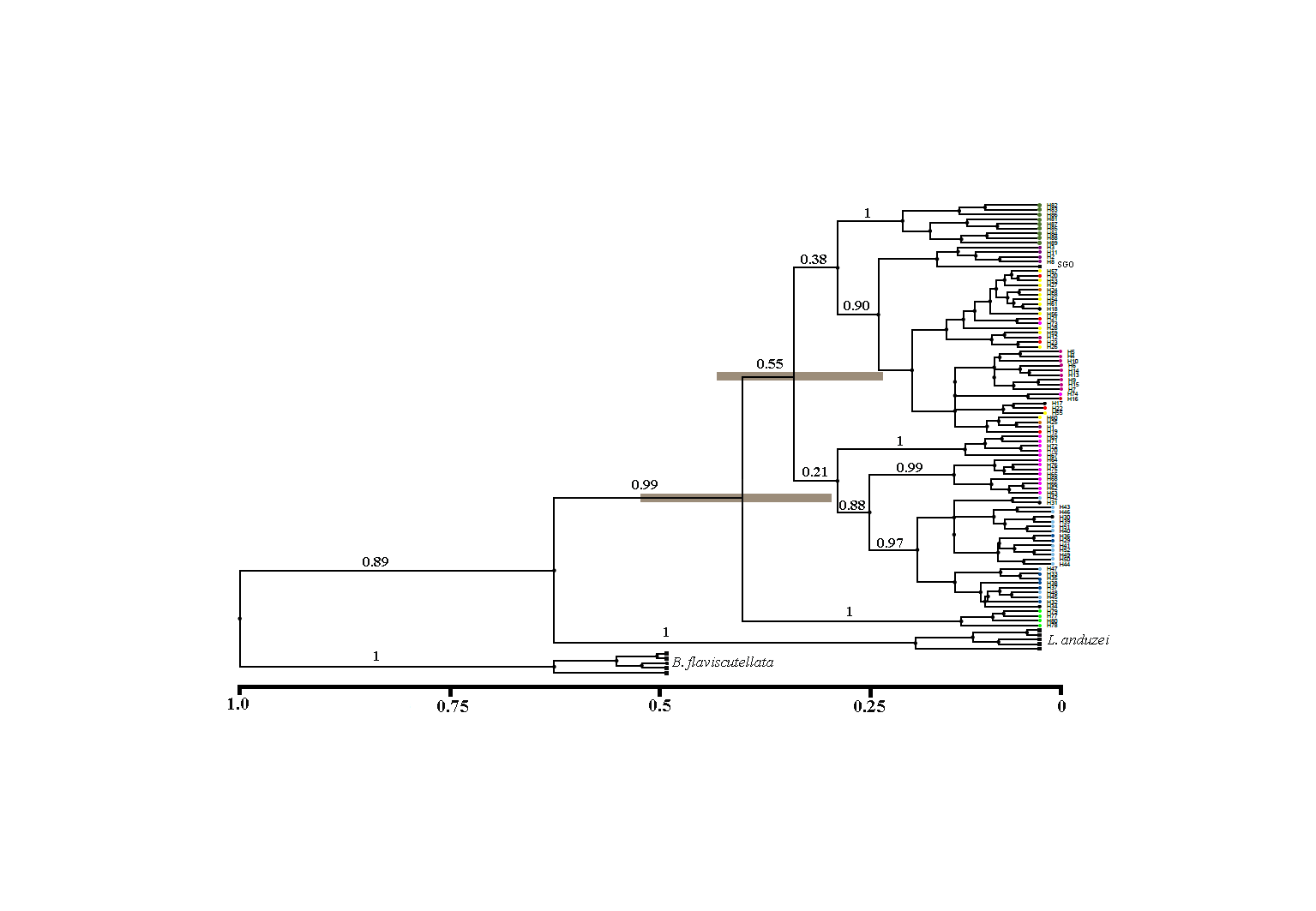

Supplement: Supplementary file 2 — Supplementary Figure S2. [file 41598_2021_93072_MOESM2_ESM.tif]

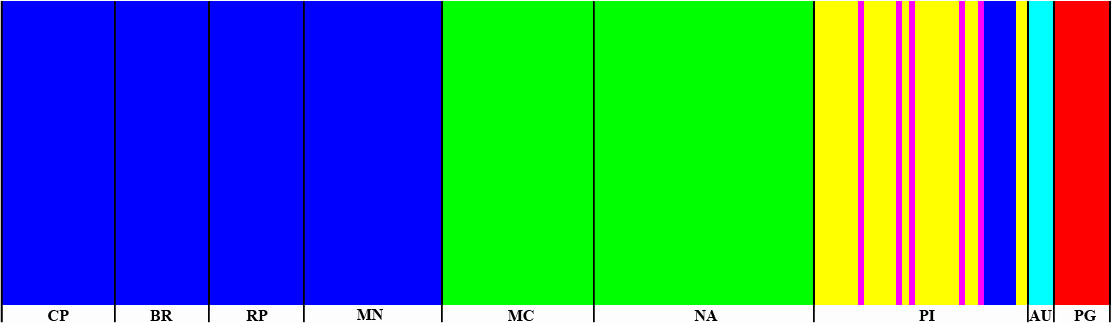

Supplement: Supplementary file 3 — Supplementary Figure S3. [file 41598_2021_93072_MOESM3_ESM.tif]

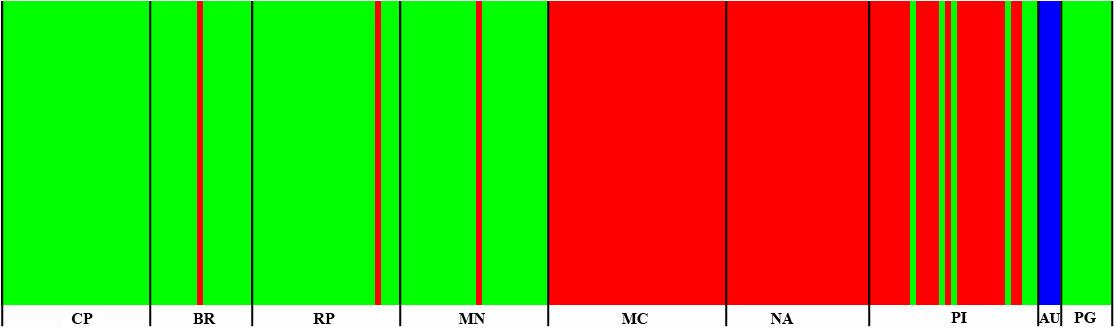

Supplement: Supplementary file 4 — Supplementary Figure S4. [file 41598_2021_93072_MOESM4_ESM.tiff]
