# Supplementary material for: Multiple evolutionary lineages for the main vector of Leishmania guyanensis, Lutzomyia umbratilis (Diptera: Psychodidae), in the Brazilian Amazon
Source: Sci Rep. 2021 Jul 28;11:15323. doi: 10.1038/s41598-021-93072-4 (PMC8319306; doi:10.1038/s41598-021-93072-4)
Supplement: Supplementary file 5 — Supplementary Information. [file 41598_2021_93072_MOESM5_ESM.docx]

**Supplementary materials**

**Table S1.** Haplotype frequency observed for the *COI* and *Cytb* genes in the *Lutzomyia umbratilis* populations from the Brazilian Amazon.

**Table S2.** Intra-population genetic diversity and neutrality tests inferred for each population of *Lutzomyia umbratilis* from the Brazilian Amazon, based on the *COI* and *Cytb* genes.

**Table S3.** Genetic differentiation among populations of *Lutzomyia umbratilis* from the Brazilian Amazon, based on the *COI* and *Cytb* genes.

**Fig. S1.** Maximum Likelihood (ML) tree generated based on the 89 haplotypes of *COI*. Tree inferred with the General Time Reversible (GTR) + G + I nucleotide substitution model and 2,000 replications. The bootstrapping support values are indicated above of the branches. The colors on the terminal branches of the tree represent the haplotypes observed in each locality, following the same color pattern of the Figures 1, 2 and 3. The black color on the terminal branches of tree represent the shared haplotypes between the localities. See Table S1 for identification of the haplotypes. Saint Georges l’ Oyapock. *Lutzomyia anduzei* and *Bichromomyia flaviscutellata* were used as outgroups.

**Fig. S2.** Bayesian Inference (BI) tree generated in *BEAST, based on the 89 haplotypes of *COI*, with the estimates of the divergence time (Mya). The colors on the terminal branches of the tree represent the haplotypes observed in each locality, following the same color pattern of the Figures 1, 2 and 3. The black color on the terminal branches (round shape) of tree represent the shared haplotypes between the localities. See Table S1 for identification of the haplotypes. SGO: Saint Georges l’ Oyapock (square shape in black color). *Lutzomyia anduzei* and *Bichromomyia flaviscutellata* were used as outgroups.

**Fig. S3.** Bayesian analysis of population structure (BAPS) of the nine *Lutzomyia umbratilis* populations from the Brazilian Amazon, using *COI* gene. Dataset analysis obtained from 176 specimens indicated the existence of six genetic groups. CP: Cachoeira Porteira; BR: BR-174 Highway; RP: Rio Preto da Eva; MN: Manaus; MC: Manacapuru; NA: Novo Airão; PI: Pitinga; AU: Autazes; PG: Porto Grande/Serra do Navio. Group 1 (blue) comprises the populations of the CP, BR, RP, MN and part of PI; Group 2 (green) represents the populations of the MC and NA; Groups 3 and 4 (yellow and pink, respectively) comprises the population of PI; Group 5 (light blue) represents the population of AU; Group 6 (red) represents the population of PG.

**Fig. S4.** Bayesian analysis of population structure (BAPS) of the nine *Lutzomyia umbratilis* populations from the Brazilian Amazon, using *Cytb* gene. Dataset analysis obtained from 187 specimens indicated the existence of three genetic groups. CP: Cachoeira Porteira; BR: BR-174 Highway; RP: Rio Preto da Eva; MN: Manaus; MC: Manacapuru; NA: Novo Airão; PI: Pitinga; AU: Autazes; PG: Porto Grande/Serra do Navio. Group 1 (green) comprises the populations of the CP, BR, RP, MN, PG; Group 2 (red) represents the populations of the MC and NA; Group 3 (blue) comprises the populations of AU. Population of Pitinga shows a mixed from Groups 1 and 2.
