# Supplementary material for: Multiple evolutionary lineages for the main vector of Leishmania guyanensis, Lutzomyia umbratilis (Diptera: Psychodidae), in the Brazilian Amazon
Source: Sci Rep. 2021 Jul 28;11:15323. doi: 10.1038/s41598-021-93072-4 (PMC8319306; doi:10.1038/s41598-021-93072-4)
Supplement: Supplementary file 6 — Supplementary Table S1. [file 41598_2021_93072_MOESM6_ESM.docx]

**Table S1.** Haplotype frequency observed for the *COI* and *Cytb* genes in the *Lutzomyia umbratilis* samples from the Brazilian Amazon.

| Gene/Locality | Haplotype frequency |
| --- | --- |
| *COI* |  |
| Cachoeira Porteira^*^ | H1(4), H2(1), H3(1), H4(1), H5(1), H6(1), H7(1), H8(1), H9(1), H10(1), H11(1), H12(1), H13(1), H14(1), H15(1) |
| BR-174 Highway^*^ | H1(5), H16(1), H17(2), H18(2), H19(1), H20(1), H21(1), H22(1), H23(1) |
| Rio Preto da Eva^*^ | H1(10), H17(3), H24(1), H25(1) |
| Manaus^**^ | H1(7), H18(3), H26(1), H27(1), H28(1), 53(1), H54(1), H55(1), H56(1), H57(1), H58(1), H59(1), H60(1), H61(1) |
| Manacapuru^*^ | H29(1), H30(2), H31(8), H32(1), H33(1), H34(7), H35(1), H36(1), H37(1), H38(1) |
| Novo Airão^*^ | H30(2), H31(12), H34(3), H39(5), H40(1), H41(1), H42(1), H43(1), H44(1), H45(1), H46(1), H47(1), H48(1), H49(1), H50(1), H51(1), H52(1) |
| Pitinga | H18(3), H62(13), H63(1), H64(1), H65(4), H66(1), H67(1), H68(2), H69(1), H70(1), H71(1), H72(1), H73(1), H74(1), H75(1), H76(1) |
| Autazes | H77(1), H78(1), H79(1), H80(1) |
| Porto Grande/Serra do Navio | H81(1), H82(1), H83(1), H84(1), H85(1), H86(1), H87(1), H88(1), H89(1) |
| ***Cytb*** |  |
| Cachoeira Porteira | H1(2), H2(1), H3(16), H4(1), H5(1), H6(1), H7(1), H8(1), H9(1), |
| BR-174 Highway | H3(13), H10(1), H11(1), H12(1), H13(1) |
| Rio Preto da Eva | H1(1), H3(20), H10(1), H14(1), H15(1), H16(1), |
| Manaus | H3(19), H15(1), H17(1), H18(1), H19(1), H20(1), H21(1) |
| Manacapuru | H22(27), H33(1), H34(2) |
| Novo Airão | H22(22), H35(1), H36(1) |
| Pitinga | H3(2), H8(1), H22(17), H23(5), H24(1), H25(1), H26(1), H27(1) |
| Autazes | H28(2), H29(1) |
| Porto Grande/Serra do Navio | H3(3), H20(1), H30(3), H31(1), H32(1) |

Inside the parentheses is the number of individuals observed for each haplotype. ^*^, samples analyzed by Scarpassa and Alencar^40^; ^**^, sample size was enlarged and re-analyzed in the present study. The *COI* haplotypes from H1 to H52 were described by Scarpassa and Alencar^40^ and the *COI* haplotypes from H53 to H89 were described in the present study. The underlined haplotypes were shared between localities.
