# Supplementary material for: Multiple evolutionary lineages for the main vector of Leishmania guyanensis, Lutzomyia umbratilis (Diptera: Psychodidae), in the Brazilian Amazon
Source: Sci Rep. 2021 Jul 28;11:15323. doi: 10.1038/s41598-021-93072-4 (PMC8319306; doi:10.1038/s41598-021-93072-4)
Supplement: Supplementary file 7 — Supplementary Table S2. [file 41598_2021_93072_MOESM7_ESM.docx]

**Table S2. Intra-population genetic diversity and neutrality tests inferred for each population of *Lutzomyia umbratilis* from the Brazilian Amazon,**

**based on the *COI* and *Cytb* genes.**

| Gene/Locality | Ts/Tv | *NS* | *K* | *h* ± SD | *π* ± SD | Tajima’s *D* | Fu’s *F*s |
| --- | --- | --- | --- | --- | --- | --- | --- |
| ***COI*** |  |  |  |  |  |  |  |
|  |  |  |  |  |  |  |  |
| Cachoeira Porteira**^#^** | 27/2 | 29 | 4.58 | 0.961 ± 0.039 | 0.00389 ± 0.00054 | -1.833* | -8.620*** |
| BR-174 Highway**^#^** | 10/0 | 10 | 2.02 | 0.886 ± 0.069 | 0.00171 ± 0.00029 | -1.307 | -4.443* |
| Rio Preto da Eva**^#^** | 5/0 | 5 | 0.88 | 0.543 ± 0.133 | 0.00074 ± 0.00030 | -1.451 | -0.626 |
| Manaus**^##^** | 13/2 | 15 | 2.22 | 0.896 ± 0.056 | 0.00188 ± 0.00023 | -1.656 | -9.940*** |
| Manacapuru**^#^** | 9/2 | 11 | 1.66 | 0.819 ± 0.057 | 0.00141 ± 0.00025 | -1.479 | -4.820* |
| Novo Airão**^#^** | 18/1 | 19 | 1.76 | 0.866 ± 0.048 | 0.00149 ± 0.00020 | -2.086* | -13.945*** |
| Pitinga | 27/4 | 31 | 7.11 | 0.843 ± 0.057 | 0.00602 ± 0.00108 | -0.219 | -1.703 |
| Autazes | 7/0 | 7 | 3.50 | 1.000 ± 0.177 | 0.00296 ± 0.00066 | -0.817 | -1.012 |
| Porto Grande/Serra do Navio | 23/5 | 28 | 8.03 | 1.000 ± 0.052 | 0.00680 ± 0.00059 | -1.104 | -3.389* |
| **Total** | **139/16** | **155** | **8.52** | **0.955 ± 0.008** | **0.00722 ± 0.00037** | **-1.742** | **-76.843***** |
|  |  |  |  |  |  |  |  |
|  |  |  |  |  |  |  |  |
| ***Cytb*** |  |  |  |  |  |  |  |
|  |  |  |  |  |  |  |  |
| Cachoeira Porteira | 10/1 | 11 | 1.03 | 0.597 ± 0.115 | 0.00201 ± 0.00053 | -2.172* | -5.770* |
| BR-174 Highway | 4/0 | 4 | 0.47 | 0.426 ± 0.147 | 0.00092 ± 0.00036 | -1.843* | -3.400* |
| Rio Preto da Eva | 5/1 | 6 | 0.48 | 0.367 ± 0.122 | 0.00094 ± 0.00037 | -2.087* | -4.371* |
| Manaus | 4/2 | 6 | 0.48 | 0.430 ± 0.124 | 0.00094 ± 0.00031 | -2.087* | -6.138* |
| Manacapuru | 2/0 | 2 | 0.20 | 0.191 ± 0.093 | 0.00038 ± 0.00019 | -1.255 | -1.669 |
| Novo Airão | 2/0 | 2 | 1.67 | 0.163 ± 0.099 | 0.00033 ± 0.00020 | -1.515 | -2.078 |
| Pitinga | 6/4 | 10 | 1.84 | 0.638 ± 0.092 | 0.00360 ± 0.00084 | -0.882 | -1.639 |
| Autazes | 1/0 | 1 | 0.67 | 0.667 ± 0.314 | 0.00130 ± 0.00061 | NC | 0.201 |
|  |  |  |  |  |  |  |  |
| Porto Grande/Serra do Navio | 6/0 | 6 | 1.61 | 0.833 ± 0.098 | 0.00315 ± 0.00081 | -1.179 | -1.338 |
| **Total** | **40/8** | **48** | **1.78** | **0.724 ± 0.023** | **0.00347 ± 0.00027** | **-2.223*** | **-34.357***** |
|  |  |  |  |  |  |  |  |
|  |  |  |  |  |  |  |  |

Ts/Tv = transitions/transversions; NS = number of segregating sites; K = average number of nucleotide differences; *h* ± SD and *π* ± SD = haplotype and

nucleotide diversities, respectively, with the respective standard deviations (SD); **^#^ =** samples analyzed by Scarpassa and Alencar^40^; **^##^** = sample size

was enlarged and re-analyzed in the present study. * = *P* < 0.05; *** = *P* < 0.001. NC = not calculated.
