# Supplementary material for: Multiple evolutionary lineages for the main vector of Leishmania guyanensis, Lutzomyia umbratilis (Diptera: Psychodidae), in the Brazilian Amazon
Source: Sci Rep. 2021 Jul 28;11:15323. doi: 10.1038/s41598-021-93072-4 (PMC8319306; doi:10.1038/s41598-021-93072-4)
Supplement: Supplementary file 8 — Supplementary Table S3. [file 41598_2021_93072_MOESM8_ESM.docx]

**Table S3.** **Genetic differentiation among populations of *Lutzomyia umbratilis* from the Brazilian Amazon, based on the *COI* and *Cytb* genes.**

| Populations/Comparisons (distance in km) | Genes | *F*_ST_ (*N*m) | *K* | *D*_xy_ | *D*_a_ | *S*_s_ | *S*_f_ |
| --- | --- | --- | --- | --- | --- | --- | --- |
| Cachoeira Porteira vs. Km 43 BR-174 (368.40) | *COI^#^* | 0.0522 (9.09) | 3.52 | 0.00297 | 0.00017 | 4 | 0 |
|  | *Cytb* | 0.0016 (309) | 0.75 | 0.00148 | 0.00001 | 0 | 0 |
| Cachoeira Porteira vs. Rio Preto da Eva (353.67) | *COI^#^* | 0.0569 (8.28) | 2.99 | 0.00248 | 0.00017 | 2 | 0 |
|  | *Cytb* | 0.0004 (1412) | 0.75 | 0.00147 | 0.00000 | 1 | 0 |
| Cachoeira Porteira vs. Manaus (394.02) | *COI^#^* | **0.1268***** (3.44) | 3.92 | **0.00332** | **0.00025** | **1** | **0** |
|  | *Cytb* | 0.0004 (1412) | 0.75 | 0.00147 | 0.00000 | 1 | 0 |
| Cachoeira Porteira vs. Manacapuru (449.22) | *COI^#^* | **0.7100***** (0.20) | 10.19 | **0.00863** | **0.00599** | **2** | **3** |
|  | *Cytb* | **0.7787***** (0.14) | 2.62 | **0.00512** | **0.00392** | **0** | **2** |
| Cachoeira Porteira vs. Novo Airaõ (477.49) | *COI^#^* | **0.7337 ***** (0.18) | 10.55 | **0.00899** | **0.00625** | **6** | **1** |
|  | *Cytb* | **0.7680***** (0.15) | 2.60 | **0.00508** | **0.00392** | **0** | **2** |
| Cachoeira Porteira vs. Pitinga (327.57) | *COI* | **0.3730***** (0.84) | 9.60 | **0.00813** | **0.00318** | **8** | **0** |
|  | *Cytb* | **0.4485***** (0.61) | 2.63 | **0.00513** | **0.00233** | **2** | **0** |
| Cachoeira Porteira vs. Autazes (373.42) | *COI* | 0.8000 (0.12) | 21.81 | 0.01846 | 0.01504 | 0 | 15 |
|  | *Cytb* | **0.8529***** (0.09) | 6.69 | **0.01307** | **0.01142** | **0** | **5** |
| Cachoeira Porteira vs. Porto Grande (639.74) | *COI* | **0.5163***** (0.47) | 12.26 | **0.01039** | **0.00505** | **5** | **2** |
|  | *Cytb* | 0.0403 (11.92) | 1.35 | 0.00263 | 0.00005 | 3 | 0 |
| Km 43 BR-174 vs. Rio Preto da Eva (30.46) | *COI^#^* | 0.0189 (25.91) | 1.48 | 0.00125 | 0.00002 | 3 | 0 |
|  | *Cytb* | -0.0102 (Inf.) | 0.47 | 0.00092 | -0.00001 | 1 | 0 |
| Km 43 BR-174 vs. Manaus (56.29) | *COI^#^* | 0.0153 (32.22) | 2.20 | 0.00187 | -0.00012 | 3 | 0 |
|  | *Cytb* | -0.0002 (Inf.) | 0.48 | 0.00093 | 0.00000 | 0 | 0 |
| Km 43 BR-174 vs. Manacapuru (87.11) | *COI^#^* | **0.8157***** (0.11) | 9.78 | **0.00833** | **0.00673** | **0** | **6** |
|  | *Cytb* | **0.8656***** (0.08) | 2.22 | **0.00433** | **0.00368** | **0** | **1** |
| Km 43 BR-174 vs. Novo Airão (107.97) | *COI^#^* | **0.8197***** (0.11) | 10.21 | **0.00869** | **0.00705** | **4** | **4** |
|  | *Cytb* | **0.8661***** (0.08) | 2.20 | **0.00430** | **0.00368** | **0** | **1** |
| Km 43 BR-174 vs. Pitinga (200.44) | *COI* | **0.4017***** (0.74) | 8.42 | **0.00713** | **0.00326** | **6** | **0** |
|  | *Cytb* | **0.4525***** (0.60) | 2.28 | **0.00446** | **0.00220** | **1** | **0** |
| Km 43 BR-174 vs. Autazes (157.90) | *COI* | **0.8899***** (0.06) | 21.05 | **0.01782** | **0.01549** | **1** | **17** |
|  | *Cytb* | 0.9245 (0.04) | 6.60 | 0.01283 | 0.01172 | 0 | 6 |
| Km 43 BR-174 vs. Porto Grande (1000.71) | *COI* | **0.6360***** (0.29) | 12.23 | **0.01036** | **0.00610** | **3** | **4** |
|  | *Cytb* | 0.1185 (3.72) | 1.12 | 0.00220 | 0.00016 | 0 | 0 |
| Rio Preto da Eva vs. Manaus (45.35) | *COI^#^* | 0.1593 (2.64) | 1.87 | 0.00158 | 0.00008 | 2 | 0 |
|  | *Cytb* | -0.0067 (Inf.) | 0.48 | 0.00093 | -0.00001 | 1 | 0 |
| Rio Preto da Eva vs. Manacapuru (96.01) | *COI^#^* | **0.8497***** (0.09) | 8.98 | **0.00765** | **0.00653** | **0** | **6** |
|  | *Cytb* | **0.8557***** (0.08) | 2.60 | **0.00441** | **0.00375** | **0** | **1** |
| Rio Preto da Eva vs. Novo Airão (130.14) | *COI^#^* | **0.8439 ***** (0.09) | 9.43 | **0.00803** | **0.00687** | **1** | **4** |
|  | *Cytb* | **0.8546** *** (0.08) | 2.24 | **0.00438** | **0.00375** | **0** | **1** |
| Rio Preto da Eva vs. Pitinga (217.50) | *COI* | **0.4162***** (0.70) | 7.86 | **0.00670** | **0.00327** | **3** | **0** |
|  | *Cytb* | **0.4870***** (0.53) | 2.32 | **0.00453** | **0.00226** | **1** | **0** |
| Rio Preto da Eva vs. Autazes (223.61) | *COI* | **0.9333***** (0.04) | 20.68 | **0.01751** | **0.01566** | **0** | **17** |
|  | *Cytb* | **0.9233***** (0.04) | 6.49 | **0.01268** | **0.01156** | **0** | **5** |
| Rio Preto da Eva vs. Porto Grande (980.00) | *COI* | **0.6820***** (0.23) | 11.74 | **0.00994** | **0.00617** | **2** | **5** |
|  | *Cytb* | **0.1319***** (3.29) | 1.12 | **0.00219** | **0.00015** | **1** | **0** |
| Manaus vs. Manacapuru (59.43) | *COI^#^* | **0.8138***** (0.11) | 10.42 | **0.00887** | **0.00699** | **0** | **7** |
|  | *Cytb* | **0.8572***** (0.08) | 2.60 | **0.00441** | **0.00375** | **0** | **1** |
| Manaus vs. Novo Airão (108.76) | *COI^#^* | **0.8198***** (0.11) | 10.84 | **0.00924** | **0.00731** | **2** | **5** |
|  | *Cytb* | **0.8546***** (0.08) | 2.24 | **0.00438** | **0.00375** | **0** | **1** |
| Manaus vs. Pitinga (253.72) | *COI* | **0.4377***** (0.64) | 8.77 | **0.00742** | **0.00347** | **7** | **0** |
|  | *Cytb* | **0.4870***** (0.53) | 2.32 | **0.00453** | **0.00226** | **1** | **0** |
| Manaus vs. Autazes (114.92) | *COI* | **0.8862***** (0.06) | 21.30 | **0.01803** | **0.01561** | **2** | **18** |
|  | *Cytb* | **0.9242***** (0.04) | 6.57 | **0.01284** | **0.01172** | **0** | **6** |
| Manaus vs. Porto Grande (1012.21) | *COI* | **0.6672***** (0.25) | 12.60 | **0.01067** | **0.0063** | **5** | **4** |
|  | *Cytb* | 0.1319 (3.30) | 1.12 | 0.00219 | 0.00015 | 1 | 0 |
| Manacapuru vs. Novo Airão (58.74) | *COI^#^* | 0.0548 (8.62) | 1.81 | 0.00153 | 0.00008 | 4 | 0 |
|  | *Cytb* | 0.0118 (41.96) | 0.18 | 0.00036 | 0.00000 | 0 | 0 |
| Manacapuru vs. Pitinga (277.74) | *COI* | **0.5173***** (0.47) | 9.49 | **0.00804** | **0.00338** | **11** | **0** |
|  | *Cytb* | **0.1559***** (2.71) | 1.20 | **0.00235** | **0.00036** | **0** | **0** |
| Manacapuru vs. Autazes (162.78) | *COI* | **0.9206***** (0.04) | 23.70 | **0.02006** | **0.01693** | **2** | **15** |
|  | *Cytb* | **0.9728***** (0.01) | 8.43 | **0.01647** | **0.01563** | **0** | **8** |
| Manacapuru vs. Porto Grande (1077.89) | *COI* | **0.7028***** (0.21) | 12.43 | **0.01052** | **0.00642** | **1** | **4** |
|  | *Cytb* | **0.8103***** (0.12) | 2.99 | **0.00584** | **0.00407** | **0** | **2** |
| Novo Airão vs. Pitinga (239.54) | *COI* | **0.5628***** (0.39) | 10.09 | **0.00854** | **0.00479** | **6** | **2** |
|  | *Cytb* | 0.1395 (3.08) | 1.19 | 0.00232 | 0.00036 | 0 | 0 |
| Novo Airão vs. Autazes (224.36) | *COI* | **0.9207***** (0.04) | 24.44 | **0.02069** | **0.01847** | **1** | **16** |
|  | *Cytb* | **0.9751***** (0.01) | 8.42 | **0.01644** | **0.01563** | **0** | **8** |
| Novo Airão vs. Porto Grande (1098.20) | *COI* | **0.7333***** (0.18) | 12.74 | **0.01078** | **0.00664** | **6** | **2** |
|  | *Cytb* | **0.7983***** (0.13) | 2.97 | **0.00581** | **0.00407** | **0** | **2** |
| Pitinga vs. Autazes (342.34) | *COI* | **0.7171***** (0.20) | 22.98 | **0.01946** | **0.01497** | **3** | **13** |
|  | *Cytb* | **0.7995***** (0.12) | 8.47 | **0.01655** | **0.01410** | **0** | **6** |
| Pitinga vs. Porto Grande (953.43) | *COI* | **0.5286***** (0.44) | 15.71 | **0.01333** | **0.00689** | **7** | **3** |
|  | *Cytb* | **0.4189***** (0.69) | 3.03 | **0.00591** | **0.00254** | **0** | **0** |
| Autazes vs. Porto Grande (958.77) | *COI* | **0.7539***** (0.16) | 26.92 | **0.02279** | **0.01791** | **1** | **18** |
|  | *Cytb* | **0.8016***** (0.12) | 7.00 | **0.01367** | **0.01145** | **0** | **5** |
|  |  |  |  |  |  |  |  |

*F*_ST =_ pair-wise genetic differentiation; *K =* average number of nucleotide differences between populations; *D*_xy =_ average number of nucleotide substitutions per site between populations; *D*_a =_ number of net nucleotide substitutions per site between populations; *S*_s =_ number of shared polymorphisms between pairs of populations; *S*_f =_ number of fixed differences between pairs of populations. **^#^ =** comparisons analyzed by Scarpassa and Alencar^40^. *** = *P =* 0.00139, after Bonferroni correction. *N*m = number of migrants per generation. Inf. = Infinity.
